# Transcriptome analysis of mRNAs, lncRNAs, and miRNAs in the skeletal muscle of Tibetan chickens at different developmental stages

**DOI:** 10.3389/fphys.2023.1225349

**Published:** 2023-07-26

**Authors:** Jie Li, Chuwen Chen, Ruipeng Zhao, Jinbo Wu, Zhixiong Li

**Affiliations:** ^1^ Laboratory of Ministry of Education for Qinghai-Tibetan Plateau Animal Genetic Resource Reservation and Utilization, Southwest Minzu University, Chengdu, Sichuan, China; ^2^ College of Animal & Veterinary Sciences, Southwest Minzu University, Chengdu, Sichuan, China; ^3^ Institute of Qinghai-Tibetan Plateau, Southwest Minzu University, Chengdu, Sichuan, China; ^4^ Institute of Science and Technology of Aba Tibetan and Qiang Autonomous Prefecture, Aba Sichuan, China

**Keywords:** chicken, mRNA, lncRNA, miRNA, skeletal muscle growth and development, ceRNA

## Abstract

**Introduction:** As a valuable genetic resource, native birds can contribute to the sustainable development of animal production. Tibetan chickens, known for their special flavor, are one of the important local poultry breeds in the Qinghai–Tibet Plateau. However, Tibetan chickens have a slow growth rate and poor carcass traits compared with broilers. Although most of the research on Tibetan chickens focused on their hypoxic adaptation, there were fewer studies related to skeletal muscle development.

**Methods:** Here, we performed the transcriptional sequencing of leg muscles from Tibetan chicken embryos at E (embryonic)10, E14, and E18.

**Results:** In total, 1,600, 4,610, and 2,166 DE (differentially expressed) mRNAs, 210, 573, and 234 DE lncRNAs (long non-coding RNAs), and 52, 137, and 33 DE miRNAs (microRNAs) were detected between E10 and E14, E10 and E18, and E14 and E18, respectively. Functional prediction showed several DE mRNAs and the target mRNAs of DE lncRNAs and DE miRNAs were significantly enriched in sarcomere organization, actin cytoskeleton organization, myofibril, muscle fiber development, and other terms and pathways related to muscle growth and development. Finally, a lncRNA–miRNA–mRNA ceRNA (competing endogenous RNA) network associated with muscle growth and development, which contained 6 DE lncRNAs, 13 DE miRNAs, and 50 DE mRNAs, was constructed based on the screened DE RNAs by Gene Ontology (GO) enrichment. These DE RNAs may play a critical regulatory role in the skeletal muscle development of chickens.

**Discussion:** The results provide a genomic resource for mRNAs, lncRNAs, and miRNAs potentially involved in the skeletal muscle development of chickens, which lay the foundation for further studies of the molecular mechanisms underlying skeletal muscle growth and development in Tibetan chickens.

## 1 Introduction

The Tibetan chicken is a unique Chinese native breed that is mainly distributed in the Qinghai–Tibet Plateau. It has excellent meat quality and flavor due to the rich content of essential amino acids and flavor amino acids. Tibetan chickens exhibit characteristics such as high adaptability, resistance to extensive management, and strong disease resistance, but with the challenges of slow growth rate and low meat yield (Tang et al., 2015; Chi et al., 2020). The yield and quality of poultry mainly depend on the development of skeletal muscle (Dransfield and Sosnicki, 1999). The number of myofibers in chickens is basically fixed at the embryonic stages, and the differentiation of primary and secondary fibers is completed by 3/4 of the incubation period (Chen et al., 2013). Therefore, it is indispensable to understand global gene expression during embryonic skeletal muscle growth and development for improving the growth rate and muscle mass of chickens.

Skeletal muscle growth and development are extremely complex processes. In addition to mRNAs, plenty of ncRNAs (non-coding RNAs) were shown to be involved in skeletal muscle growth and development in multiple species, including pigs, sheep, chickens, and cattle (Cai et al., 2017; Wang et al., 2022a; Zhan et al., 2022; Zhang et al., 2022). miRNAs can inhibit post-transcriptional gene expression by binding to specific mRNAs. For example, miR-2954 prevented myoblast differentiation into multinucleated myotubes via suppressing the expression of *YY1* (YY1 transcription factor) gene in the process of chicken skeletal muscle development at the embryonic stage (Dong et al., 2021). lncRNA is highly abundant ncRNA (Guttman and Rinn, 2012) that has complex biological functions such as interfering with gene expression, interacting with proteins, acting as ceRNAs (competing endogenous RNAs), and encoding peptides (Matsumoto et al., 2017; Li et al., 2020; Wang et al., 2020; Zhang et al., 2020). In recent years, several studies have confirmed that lncRNAs are widely involved in many stages of muscle growth and development in livestock and poultry (Li et al., 2018). lncRNA-Six1 affected muscle growth and development via encoding a micropeptide (Cai et al., 2017). lncRNAs work as ceRNAs, which is the most common regulatory function. For example, lncRNA MEG3, as the “sponge” of miRNA-135, promoted bovine skeletal muscle differentiation via interacting with miRNA-135 and *MEF2C* (myocyte enhancer factor 2C) (Liu et al., 2019), and this lncRNA was also found in pig skeletal muscle (Yu et al., 2018). lncRNA-125b promoted skeletal muscle satellite cell differentiation by functioning as a ceRNA for miR-125b to positively regulate *IGF2* (insulin-like growth factor 2) expression (Zhan et al., 2019). To explore the role of coding RNAs and ncRNAs in the development of chicken skeletal muscle, we performed a systematic transcriptome analysis of mRNAs, lncRNAs, and miRNAs in the skeletal muscle of chicken embryos at various developmental stages.

For poultry, skeletal muscle development during the embryonic period plays a fateful role in the potential of post-incubation muscle development. The primary and secondary muscle fibers were formed in approximately 7–12 days, independent myotube formation and differentiation were initiated mainly from 12 to 18 days, and muscle fiber hypertrophy was observed during the post 1/4 of embryo incubation (Stockdale and Miller, 1987; Stickland et al., 2004; Picard et al., 2010). Therefore, in this study, the leg muscles from Tibetan chickens at E10-, E14-, and E18-day were selected for sequencing analysis. DE mRNAs, DE lncRNAs, and DE miRNAs were screened from three comparisons (E10 vs. E14, E10 vs. E18, and E14 vs. E18). Common DE RNAs, which were present in all three comparisons, were obtained through Venn analysis. Subsequently, Gene Ontology (GO) and Kyoto Encyclopedia of Genes and Genomes (KEGG) functional enrichment analyses were performed on common DE mRNAs, target genes of common DE lncRNAs, and common DE miRNAs. The ceRNA network contributes to exploring the functions and regulatory mechanisms of genes and facilitates a deeper understanding of many biological phenomena. So, we constructed the ceRNA regulatory networks associated with skeletal muscle growth and development. Some key factors were expected to be revealed for chicken muscle development. These findings will help us understand the regulation of coding and ncRNAs in chicken muscle growth and development.

## 2 Materials and methods

### 2.1 Animal preparation and sample collection

In this experiment, we collected eggs from Tibetan chickens (TCs) obtained from Mao Xian Jiuding Original Ecological Livestock and Poultry Breeding Co., Ltd. (Aba Tibetan and Qiang Autonomous, Sichuan Province, China). The eggs were incubated at a humidity of 55% and a temperature of 37.8°C. At the target time, we dissected the chicken embryos and determined gender by observing the gonadal features of the fetus. All leg muscle samples from nine Tibetan chickens were collected from healthy male embryos at three developmental stages (E10, E14, and E18), with three samples in each stage. All collected tissues were immediately frozen in liquid nitrogen and subsequently stored at −80°C for RNA extraction and sequencing. All treatment and sample collection procedures were approved by the IACUC (Institutional Animal Care and Use Committee) of Southwest Minzu University (Sichuan, China) with approval number 2020MDLS44.

### 2.2 Total RNA isolation, library construction, and sequencing

Total RNA was isolated from nine leg muscle samples at three differential development stages using the TRIzol reagent (Vazyme, Nanjing, China). The quality of the RNA samples was detected by 1% agarose gel electrophoresis. The purity and concentration of the RNA samples were assessed using the NanoPhotometer^®^ N60 (Implen, Munich, Germany), and the integrity and RNA integrity number (RIN) values of the isolated RNAs were evaluated using the Agilent Bioanalyzer 2100 system (Agilent Technologies, Massy, France). The RIN values of all nine samples were greater than 8, and all could be used for subsequent analysis. The isolated RNAs that passed these tests were divided into three parts. One part was used for quantitative real-time PCR (RT-qPCR) by reverse transcription into cDNA strands using the PrimeScriptTM RT Reagent Kit with gDNA Eraser (Perfect Real Time) (Takara, Dalian, China), and the other two parts were used for long RNA-seq (mRNA and lncRNA sequencing) and small RNA-seq (miRNA sequencing).

For long RNA-seq, 9 μg of total RNA in each sample was used to remove ribosomal RNA (rRNA) using the Ribo-Zero™ rRNA Removal Kit (Epicentre, Madison, WI, United States). The total RNA of nine samples (TC_E10-1, TC_E10-2, TC_E10-3, TC_E14-1, TC_E14-2, TC_E14-3, TC_E18-1, TC_E18-2, and TC_E18-3) from which rRNA was removed was used as the input material. According to the NEBNext^®^ Ultra™ Directional RNA Library Prep Kit for Illumina (NEB, United States), nine cDNA libraries were constructed from RNAs obtained in the previous step, and three biological replicates per developmental stage were prepared. Finally, the cDNA libraries were sequenced on Illumina HiSeq 2500 (Majorbio Bio-pharm Technology Co., Ltd., Shanghai, China), and 125-bp paired-end reads were generated. For small RNA-seq, approximately 3 μg of total RNA per sample (nine samples) was used for preparing the libraries following the manufacturer’s recommendations in the SMARTer smRNA-Seq Kit for Illumina (Clontech, United States). Subsequently, ligation of the RNA 3′- and 5′-adapters, reverse transcription of the synthetic first strand, PCR amplification, and PAGE gel purification were performed. The cDNA libraries for small RNA-seq were constructed. Finally, the libraries were also sequenced on Illumina HiSeq 2500, and 50-bp single-end reads were generated.

### 2.3 Data quality control and read mapping

Large volumes of raw data stored in the FASTQ format were obtained after sequencing. In order to ensure the reliability of the subsequent bioinformatics analysis, the raw data were first quality-controlled. Clean data were obtained from raw data using SeqPrep (https://github.com/jstjohn/SeqPrep) and Sickle (https://github.com/najoshi/sickle) software tools. Specific operations include removing the reads containing adapter, removing the reads containing poly-N (N rate >10%), and removing the low-quality reads (cutting the bases with quality (Q) < 20 at the end of the sequence (3′-end); if there are still bases with Q < 10 in the remaining sequence, the whole sequence will be eliminated; otherwise, it will be retained). For small RNA-seq, in addition to the aforementioned quality control operations, reads shorter than 18 nt and longer than 32 nt were filtered out. After the data quality control, the quality assessment was carried out, and the Q20, Q30, and GC content of clean data were calculated. All further analyses were carried out based on the high-quality, clean data.

For long RNA-seq and small RNA-seq, quality-qualified clean data (reads) were mapped to the chicken reference genome (the resource of the reference genome: http://asia.ensembl.org/Gallus_gallus/Info/Index, the version of the reference genome: GRCg6a) using HISAT2 (http://ccb.jhu.edu/software/hisat2/index.shtml) (Kim et al., 2019) and Bowtie2 (v2.2.9) (Langmead and Salzberg, 2012), respectively. Furthermore, the possibility of clean reads that span exons was considered, and many clean reads that failed to align were segmented for re-mapping using TopHat2 (http://ccb.jhu.edu/software/tophat/index.shtml). The mapped reads were assembled using Cufflinks (http://cole-trapnell-lab.github.io/cufflinks/) and StringTie (http://ccb.jhu.edu/software/stringtie/) software applications (Pertea et al., 2015).

### 2.4 Identification of lncRNAs

lncRNA sequences that have been previously reported were downloaded from lncRNA databases, mainly including NONCODE (http://www.noncode.org/index.php), Ensembl, and NCBI databases. Based on these databases, known lncRNAs were identified. For the identification of novel lncRNAs, the transcripts with class code “x (exonic overlap with the reference on the opposite strand),” “i (transfrag falling entirely within a reference intron),” “j (potentially novel transcript),” “u (unknown, intergenic transcript),” and “o (generic exonic overlap with a reference transcript)” were obtained using gffcompare software. On this basis, the transcripts whose length ≥200 bp, exon number ≥2, and ORF length ≤300 bp were screened as candidate lncRNAs. The encoding potential of candidate lncRNAs was screened using the analysis methods of coding potential calculator (CPC), coding-non-coding index (CNCI), coding potential assessment tool (CPAT), and Pfam protein domain analysis tools (Kong et al., 2007; Sun et al., 2013; Wang et al., 2013; Robert et al., 2014), and the screening conditions of these four methods are CPC score <0.5, CNCI score <0, CPAT score <0.5, and Pfam database without comments. The intersection of the results obtained from these four analysis methods represents the set of novel lncRNAs.

Quantitative analysis of lncRNA expression levels was performed using RSEM software (http://deweylab.github.io/RSEM/). The expression of lncRNAs was measured by TPM (transcripts per million reads). Based on the expression details, a correlation analysis between biological replicate samples for long RNA-seq was performed using the Pearson correlation algorithm.

### 2.5 Identification of miRNAs

Known miRNAs were identified by aligning clean reads to pre-miRNAs (miRNA precursors) and mature miRNAs in the miRbase database (http://www.mirbase.org/). Simultaneously, ribosomal RNAs (rRNAs), transfer RNAs (tRNAs), small nuclear RNAs (snRNAs), small nucleolar RNAs (snoRNAs), and other ncRNAs and repeats were removed, and unannotated reads were obtained. According to the particular hairpin structure of pre-miRNAs, novel miRNAs were predicted using miRDeep2 software (https://www.mdc-berlin.de/content/mirdeep2-documentation) (Friedländer et al., 2012). First, the unannotated reads were aligned with the reference genome, and the surrounding sequences were intercepted for secondary structure prediction. Next, according to the prediction results, the unannotated reads were filtered using information of the Dicer cutting site, energy values, and other features to identify novel miRNAs. The expression levels of miRNAs were also measured via TPM. Based on the expression details, a correlation analysis between biological replicate samples for small RNA-seq was performed using the Pearson correlation algorithm.

### 2.6 Screening of differentially expressed RNAs and functional prediction

Differentially expressed mRNAs, lncRNAs, and miRNAs were analyzed using DESeq2, DEGseq, and edgeR software programs, for which the filter criteria are *P*_*adj*_ [corrected *p*-value by BH (fdr correction with Benjamini/Hochberg)] < 0.05, *P*_*adj*_ < 0.001, and *P*_*adj*_ < 0.05, respectively, and |log_2_(fold change)| ≥ 1 (Love et al., 2014).

The lncRNA target genes of cis-acting were predicted by satisfying the requirement that the lncRNA affects the expression of surrounding genes (within 100 kb). When the expression of lncRNAs is significantly positively or negatively correlated with the expression of some genes, the lncRNA target genes of trans-acting were predicted by the correlation analysis of expression between lncRNAs and protein-encoding genes. The prediction of miRNA target genes was carried out using miRanda, TargetScan, and RNAhybrid software applications (Lewis et al., 2003; Betel et al., 2008).

GO (http://www.geneontology.org/) analyses were carried out for DE mRNAs, the target genes of DE lncRNAs, and DE miRNAs using GOATOOLS software. Furthermore, KEGG (http://www.genome.jp/kegg/) pathway enrichment analyses were performed for DE mRNAs, the target genes of DE lncRNAs, and DE miRNAs by Majorbio Bio-pharm Technology Co., Ltd. The terms and pathways with *P*_*adj*_ < 0.05 were regarded as significant enrichments.

### 2.7 Construction of the ceRNA (lncRNA–miRNA–mRNA) network

CeRNAs are important and novel transcriptional regulators. The ceRNA network is one of the most important methods for post-transcriptional regulation of non-coding RNAs (Braga et al., 2020). The interactions of DE miRNA–DE mRNA and DE miRNA–DE lncRNA related to skeletal muscle growth and development were predicted using miRanda, TargetScan, and RNAhybrid software applications. Based on the ceRNA network theory, Cytoscape software (v3.6.0) was used to construct and visualize the ceRNA (lncRNA–miRNA–mRNA) network (Poliseno et al., 2010; Salmena et al., 2011).

### 2.8 Validation of DE RNAs by RT-qPCR

To verify the accuracy and reliability of differentially expressed RNAs (DE RNAs) screened by RNA-seq, six DE mRNAs, six DE lncRNAs, and six DE miRNAs were randomly selected for RT-qPCR. We used the PrimeScript™ RT Reagent Kit with gDNA Eraser (Perfect Real Time) (Takara, Dalian, China) to convert RNAs to cDNAs. The random hexamers were used for DE mRNAs and DE lncRNAs, and stem-loop RT primers were used for DE miRNAs. RT-qPCR was performed using the TB Green^®^ Premix Ex Taq™Ⅱ Kit (Takara, Dalian, China) according to the manufacturer’s instructions. *GAPDH* for mRNAs and lncRNAs and *U6* for miRNAs were used as endogenous controls. All primers used in RT-qPCR are designed using Primer Premier 5 software and exhibited in Supplementary Data S1. RT-qPCR was carried out in the LightCycler480 system (Roche, Swiss Confederation) with three replicates for each sample. The data were expressed as values relative to the E10 group. The relative expression levels of DE mRNAs, DE lncRNAs, and DE miRNAs were calculated using the 2^−ΔΔCT^ method, and one-way ANOVA in SPSS 20.0 was used to analyze the data. Differences were considered significant at *p* < 0.05.

All tools, databases, and related information used in this study are shown in Supplementary Data S2.

## 3 Results

### 3.1 Overview of RNA-seq data of Tibetan chicken leg muscle at different embryonic stages

After the quality control for raw reads, a total of 1,051,297,502 clean reads for long RNA-seq and 97,422,413 clean reads for small RNA-seq were obtained from all samples. The percentages of Q20 and Q30 were above 97.8% and 93.91%, respectively. The average GC content of all samples was approximately 45.71%, and the average base error rate was below 0.0227% (Supplementary Data S3). The clean reads were mapped to the chicken reference genome with a range of 91.24%–94.68% for long RNA-seq and 96.32%–99.09% for small RNA-seq (Supplementary Data S4).

**FIGURE 1:**
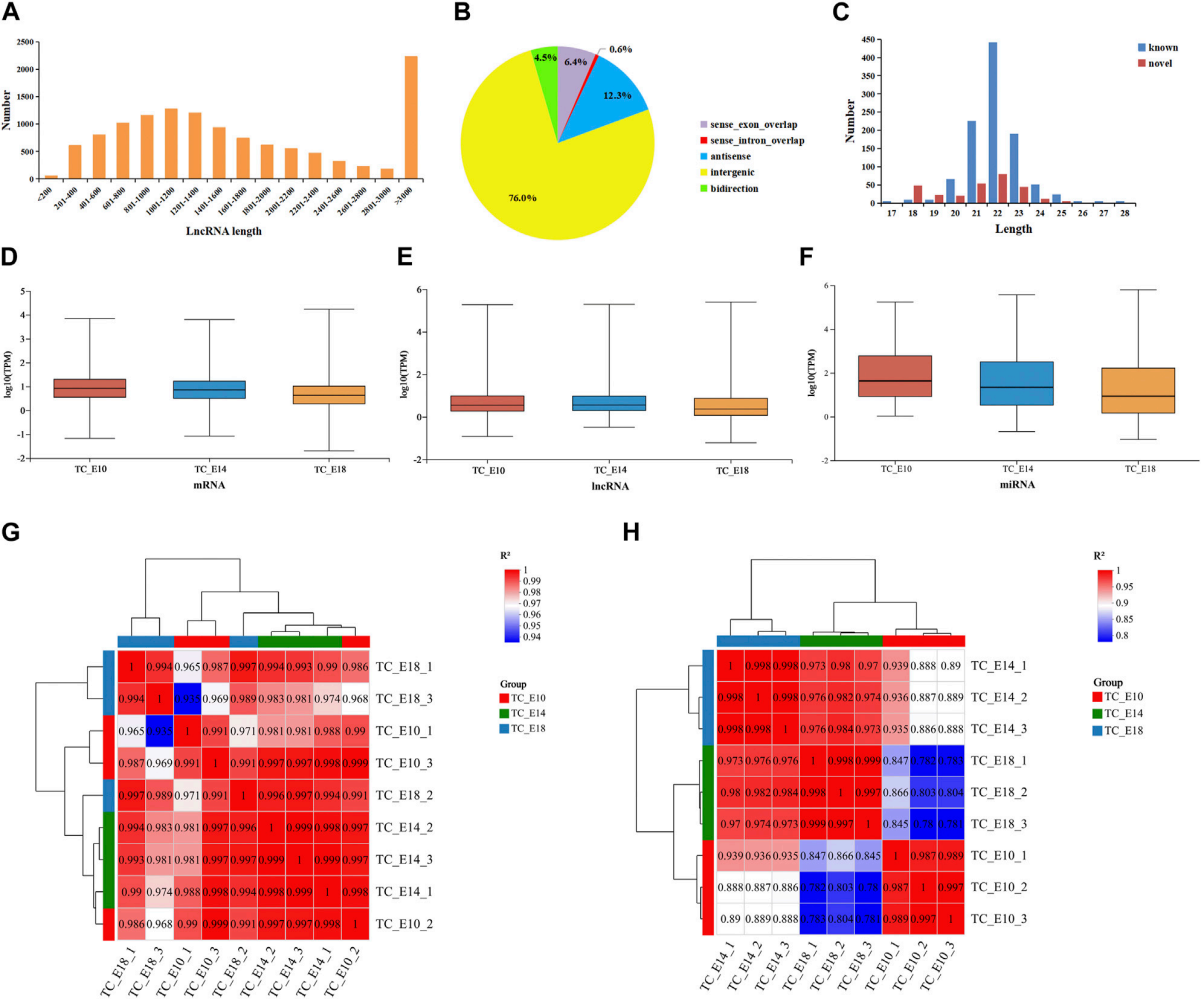
Features of RNAs in the leg muscles of Tibetan chicken embryos. Length distribution **(A)** and classification statistics **(B)** of lncRNAs. **(C)** Length distribution of miRNAs. Distribution of mRNA **(D)**, lncRNA **(E)**, and miRNA **(F)** expression values (TPM) of three different developmental stages containing nine samples. Inter-sample correlation analysis for long RNA-seq **(G)** and small RNA-seq **(H)**.

At the three development stages, there are 29,615 mRNAs, 12,399 lncRNAs (containing 11,014 known and 1,385 novel lncRNAs), and 1,216 miRNAs (containing 944 known and 272 novel miRNAs). The length of lncRNAs was mainly longer than 3,000 bp (Figure 1A), and the majority of the lncRNAs were intergenic-lncRNAs (76.0%), followed by antisense-lncRNAs (12.3%), sense-exon-overlap lncRNAs (6.4%), bidirectional-lncRNAs (4.5%), and sense-intron-overlap lncRNAs (0.6%) (Figure 1B). According to the characteristics of miRNAs, the length of miRNAs ranged from 17 to 28 bp, and most of the miRNAs (known and novel miRNAs) were between 20 and 24 bp long (Figure 1C). The distribution of TPM values for mRNAs, lncRNAs, and miRNAs was found to be similar across the three different developmental stages in Tibetan chicken leg muscles (Figures 1D–F). The expression levels of mRNAs, lncRNAs, and miRNAs were concentrated, and the results of inter-sample correlation analysis for long RNA-seq (Figure 1G) and small RNA-seq (Figure 1H) were valid (the correlation coefficients between the biological replicates were above 0.98), which suggested that these RNA-seq results are reliable for further analysis.

### 3.2 Functional prediction of differentially expressed RNAs between E10 and E14

**FIGURE 2:**
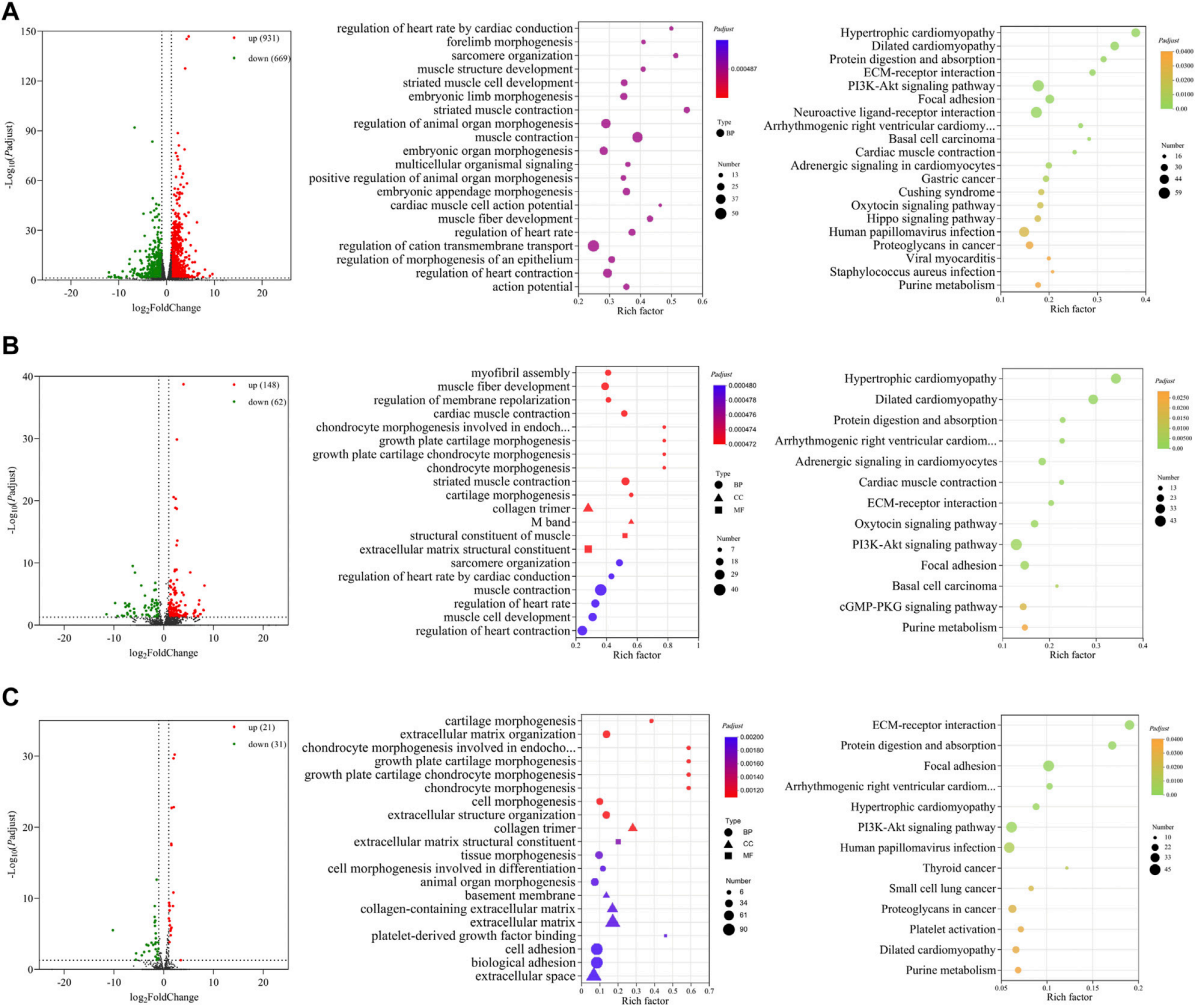
Functional prediction of DE RNAs between E10 and E14. **(A)** Volcano plot and GO and KEGG enrichment analyses of DE mRNAs. **(B)** Volcano plot of DE lncRNAs and GO and KEGG enrichment analyses of target mRNAs of DE lncRNAs. **(C)** Volcano plot of DE miRNAs and GO and KEGG enrichment analyses of target mRNAs of DE miRNAs. Red points represent upregulation, and green points represent downregulation in volcano plots. GO and KEGG bubble charts show the significantly enriched top 20 terms and pathways.

Three comparisons (E10 vs. E14, E10 vs. E18, and E14 vs. E18) between E10, E14, and E18 were performed during the skeletal muscle growth and development of Tibetan chickens. In E10 vs. E14, 1,600 DE mRNAs (Figure 2A), 210 DE lncRNAs (Figure 2B), and 52 DE miRNAs (Figure 2C) were found, among which 931 DE mRNAs, 148 DE lncRNAs, and 21 DE miRNAs were upregulated and 669 DE mRNAs, 62 DE lncRNAs, and 31 DE miRNAs were downregulated (Supplementary Data S5). The top 20 upregulated and downregulated mRNAs, lncRNAs, and miRNAs based on log_2_(fold change) in E10 vs. E14 are summarized in Table 1 and Supplementary Data S6.

**TABLE 1:** Top 20 upregulated and downregulated mRNAs in the comparison group E10 vs. E14.

Category	Gene ID	Gene name	Log_2_(fold change)	*P*_adjust_
Upregulated mRNAs	ENSGALG00000054492	—	9.589327327	2.40E-04
	ENSGALG00000051623	—	9.059631062	2.95E-03
	ENSGALG00000050210	—	8.016877356	4.49E-07
	ENSGALG00000047672	—	7.626353826	1.60E-02
	ENSGALG00000054721	—	6.842710879	8.04E-05
	ENSGALG00000041066	*DNASE1*	6.680642271	7.05E-05
	ENSGALG00000054020	—	6.680306341	1.75E-04
	ENSGALG00000016620	*PNOC*	6.507333036	9.97E-05
	ENSGALG00000048599	—	6.410608364	2.03E-03
	ENSGALG00000045362	—	6.357002439	1.32E-35
Downregulated mRNAs	ENSGALG00000014184	*HINTW*	−12.08661526	8.43E-03
	ENSGALG00000035998	—	−12.04318636	2.06E-05
	ENSGALG00000048542	—	−11.97928931	9.14E-03
	ENSGALG00000040263	—	−11.58814176	9.11E-03
	ENSGALG00000031327	—	−10.86353055	1.31E-03
	ENSGALG00000041221	—	−10.81500874	2.05E-02
	ENSGALG00000038064	—	−10.59823748	2.36E-02
	ENSGALG00000040780	*UBAP2*	−10.59268645	2.36E-02
	ENSGALG00000040704	*SPIN1W*	−10.10320139	1.23E-02
	ENSGALG00000047904	—	−9.929654764	3.57E-02

GO and KEGG functional enrichment analyses were carried out with DE mRNAs and target mRNAs of DE lncRNAs and DE miRNAs between E10 and E14. Significantly enriched GO terms and KEGG pathways (*P*_*adj*_ ≤ 0.05) of the top 20 are displayed in Figures 2A–C. In total, 1,600 DE mRNAs are mainly enriched in GO terms such as sarcomere organization, muscle structure development, embryonic limb morphogenesis, muscle contraction, and muscle fiber development. The KEGG enrichment analysis revealed the involvement of DE mRNAs in extracellular matrix (ECM)–receptor interaction, PI3K-Akt signaling pathway, and other pathways (Figure 2A).

A total of 1,173 cis- and trans-target mRNAs were predicted for 210 DE lncRNAs between E10 and E14. The target mRNAs are mainly enriched in GO terms such as myofibril assembly, muscle fiber development, M band, structural constituent of muscle, sarcomere organization, and muscle cell development. Enriched KEGG pathways also included ECM–receptor interaction and PI3K-Akt signaling pathway; in addition, there are many target mRNAs enriched in the cGMP-PKG signaling pathway (Figure 2B). For 52 DE miRNAs, we predicted 997 target mRNAs that were enriched in the top 20 GO terms including collagen-containing extracellular matrix, collagen trimer, and regulation of embryonic development. A total of 13 significantly enriched KEGG pathways were obtained from the target mRNAs of DE miRNAs, which mainly included ECM–receptor interaction, PI3K-Akt signaling pathway, and other pathways related to the regulation of diseases (Figure 2C).

### 3.3 Functional prediction of differentially expressed RNAs between E10 and E18

**FIGURE 3:**
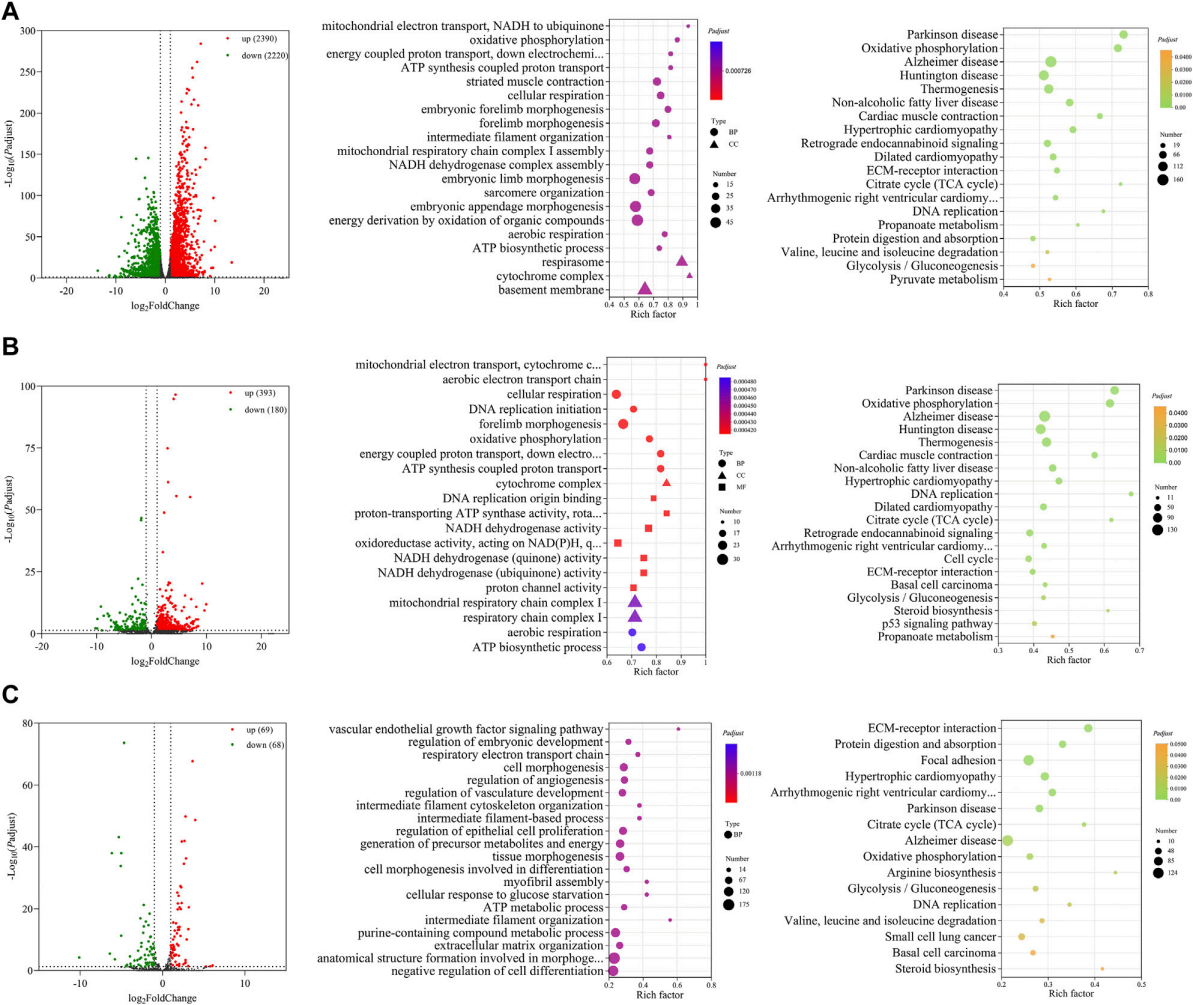
Functional prediction of DE RNAs between E10 and E18. **(A)** Volcano plot and GO and KEGG enrichment analyses of DE mRNAs. **(B)** Volcano plot of DE lncRNAs and GO and KEGG enrichment analyses of target mRNAs of DE lncRNAs. **(C)** Volcano plot of DE miRNAs and GO and KEGG enrichment analyses of target mRNAs of DE miRNAs. Red points represent upregulation, and green points represent downregulation in volcano plots. GO and KEGG bubble charts show the significantly enriched top 20 terms and pathways.

A total of 4,610 DE mRNAs (Figure 3A), 573 DE lncRNAs (Figure 3B), and 137 DE miRNAs (Figure 3C) were identified in the comparison group E10 vs. E18. Of these, 2,390 DE mRNAs, 393 DE lncRNAs, and 69 DE miRNAs were upregulated, and 2,220 DE mRNAs, 180 DE lncRNAs, and 68 DE miRNAs were downregulated (Supplementary Data S7). The top 20 upregulated and downregulated mRNAs, lncRNAs, and miRNAs based on log_2_(fold change) in E10 vs. E18 are summarized in Table 2 and Supplementary Data S8.

**TABLE 2:** Top 20 upregulated and downregulated mRNAs in the comparison group E10 vs. E18.

Category	Gene ID	Gene name	Log_2_(fold change)	*P*_adjust_
Upregulated mRNAs	ENSGALG00000021552	*RASL10A*	13.44117478	1.08E-19
	ENSGALG00000015602	*CKMT2*	10.09521992	5.48E-70
	ENSGALG00000016620	*PNOC*	9.974697488	1.58E-38
	ENSGALG00000013634	*LRRC2*	9.724774963	7.35E-98
	ENSGALG00000029212	*PADI2*	9.638699637	2.96E-13
	ENSGALG00000032845	*ASB10*	9.312053494	5.00E-13
	ENSGALG00000045362	—	9.259685132	0
	ENSGALG00000007539	*ANKRD2*	9.217837837	1.58E-34
	ENSGALG00000029526	—	9.047692013	4.10E-03
	ENSGALG00000034427	*SCN4A*	8.152985463	1.16E-144
Downregulated mRNAs	ENSGALG00000035998	*HINTW*	−13.65866032	1.17E-09
	ENSGALG00000014184		−11.42568727	8.11E-03
	ENSGALG00000040704	*SPIN1W*	−11.35741971	8.53E-03
	ENSGALG00000040263		−11.31948341	3.30E-04
	ENSGALG00000048542		−11.31837931	8.78E-03
	ENSGALG00000031327		−10.20238068	1.44E-03
	ENSGALG00000041221		−10.15402646	2.01E-02
	ENSGALG00000038064		−9.937155237	2.32E-02
	ENSGALG00000014994	*CRHBP*	−9.37034238	1.19E-26
	ENSGALG00000047904		−9.268718459	3.57E-02

For 4,610 DE mRNAs, significantly enriched GO terms (*P*_*adj*_ ≤ 0.05) in the top 20 mainly contained striated muscle contraction, embryonic forelimb morphogenesis, intermediate filament organization, sarcomere organization, and ATP biosynthetic process. The significantly enriched KEGG pathways were the citrate cycle (TCA cycle), DNA replication, valine, leucine, and isoleucine degradation, glycolysis/gluconeogenesis, and some pathways associated with diseases (Figure 3A).

A total of 3,474 cis- and trans-target mRNAs of 573 DE lncRNAs were mainly enriched in the top 20 GO terms, including DNA replication initiation, cellular respiration, forelimb morphogenesis, cytochrome complex, DNA origin binding, NADH dehydrogenase activity, and ATP biosynthetic process. In addition, the enriched KEGG pathways were mainly TCA cycle, cell cycle, ECM–receptor interaction, glycolysis/gluconeogenesis, steroid biosynthesis, and p53 signaling pathway (Figure 3B). For 137 DE miRNAs, we predicted 4,678 target mRNAs that were mainly involved in GO terms such as myofibril assembly, regulation of vasculature development, regulation of embryonic development, and intermediate filament organization. These target mRNAs were significantly enriched in the KEGG pathways of ECM–receptor interaction, TCA cycle, arginine biosynthesis, valine, leucine, and isoleucine degradation, steroid biosynthesis, and glycolysis/gluconeogenesis (Figure 3C).

### 3.4 Functional prediction of differentially expressed RNAs between E14 and E18

**FIGURE 4:**
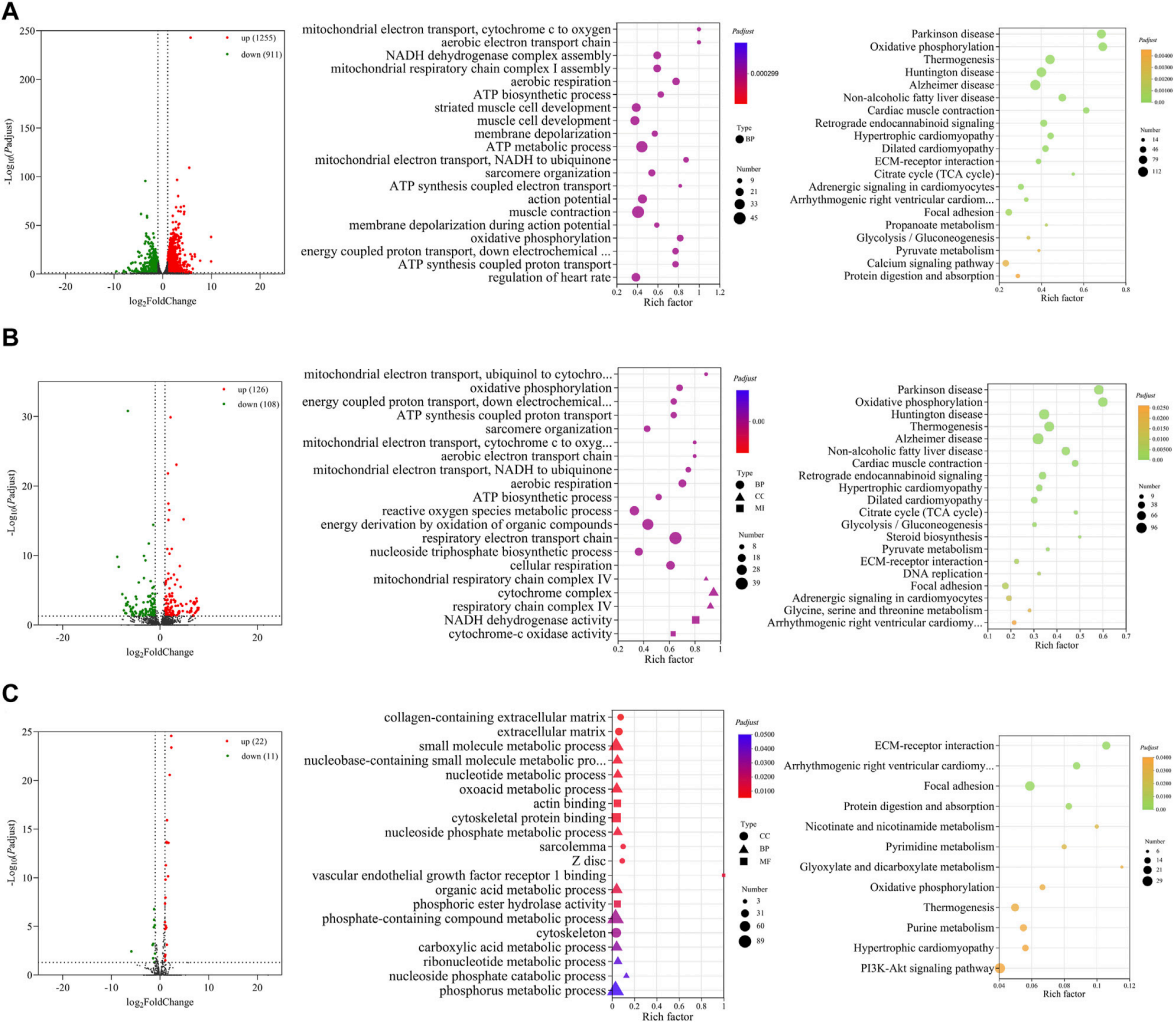
Functional prediction of DE RNAs between E14 and E18. **(A)** Volcano plot and GO and KEGG enrichment analyses of DE mRNAs. **(B)** Volcano plot of DE lncRNAs and GO and KEGG enrichment analyses of DE target mRNAs of lncRNAs. **(C)** Volcano plot of DE miRNAs and GO and KEGG enrichment analyses of target mRNAs of DE miRNAs. Red points represent upregulation, and green points represent downregulation in volcano plots. GO and KEGG bubble charts show the significantly enriched top 20 terms and pathways.

In comparison between E14 and E18, we screened 2,166 DE mRNAs (1,255 upregulation and 911 downregulation), 234 DE lncRNAs (126 upregulation and 108 downregulation), and 33 DE miRNAs (22 upregulation and 11 downregulation) (Figures 4A–C; Supplementary Data S9). The top 20 upregulated and downregulated mRNAs, lncRNAs, and miRNAs based on log_2_(fold change) in E14 vs. E18 are summarized in Table 3; Supplementary Data S10.

**TABLE 3:** Top 20 upregulated and downregulated mRNAs in the comparison group E14 vs. E18.

Category	Gene ID	Gene name	Log_2_(fold change)	*P*_adjust_
Upregulated mRNAs	ENSGALG00000003179	*ANGPTL7*	9.927928964	1.36E-13
	ENSGALG00000021552	*RASL10A*	9.91220815	9.62E-39
	ENSGALG00000046548	—	7.683724972	2.20E-14
	ENSGALG00000029212	*PADI2*	6.598843583	1.06E-18
	ENSGALG00000007539	*ANKRD2*	6.546802783	7.74E-21
	ENSGALG00000053885	—	6.149233691	4.20E-06
	ENSGALG00000013223	*CLEC3A*	5.986466741	1.05E-18
	ENSGALG00000039536	*C2H8ORF22*	5.951805468	5.55E-16
	ENSGALG00000049368	—	5.808190919	2.12E-02
	ENSGALG00000007114	*APOA1*	5.712611308	1.20E-243
Downregulated mRNAs	ENSGALG00000054492	—	−9.53684764	2.24E-03
	ENSGALG00000051623	—	−8.04562968	9.15E-03
	ENSGALG00000033381	—	−8.015708394	5.61E-04
	ENSGALG00000018415	—	−7.640307752	1.14E-02
	ENSGALG00000053121	—	−7.109572814	2.83E-07
	ENSGALG00000027002	—	−6.891598098	9.14E-06
	ENSGALG00000010657	—	−6.817239757	2.54E-05
	ENSGALG00000054721	—	−6.791266319	1.34E-04
	ENSGALG00000015079	*UPK1B*	−6.755609787	3.24E-05
	ENSGALG00000014994	*CRHBP*	−6.478074232	1.77E-11

Similarly, GO and KEGG functional analyses were performed. The top 20 significantly enriched GO terms and KEGG pathways of DE mRNAs are shown in Figure 4A. The GO terms mainly included ATP biosynthetic process, striated muscle cell development, muscle cell development, sarcomere organization, muscle contraction, and other terms related to cell respiration. The KEGG pathways of the regulation of diseases, TCA cycle, regulation of cardiac muscle, glycolysis/gluconeogenesis, and calcium signaling pathway were significantly enriched.

There were 1,561 cis- and trans-target mRNAs identified for 234 DE lncRNAs. Significantly enriched top 20 GO terms for the target mRNAs of DE lncRNAs primarily included sarcomere organization, ATP biosynthetic process, reactive oxygen species metabolic process, and other terms related to cell respiration. Significantly enriched top 20 KEGG pathways were primarily associated with the regulation of diseases, amino acid metabolism, TCA cycle, glycolysis/gluconeogenesis, and steroid biosynthesis (Figure 4B). We predicted 625 target mRNAs for 33 DE miRNAs. The significantly enriched GO terms were collagen-containing extracellular matrix, sarcolemma, Z disc, actin binding, and vascular endothelial growth factor receptor Ⅰ binding. Furthermore, the KEGG pathways were ECM–receptor interaction, focal adhesion, protein digestion and absorption, oxidative phosphorylation, and PI3K-Akt signaling pathway (Figure 4C).

### 3.5 Selection and functional prediction of common differentially expressed RNAs

**FIGURE 5:**
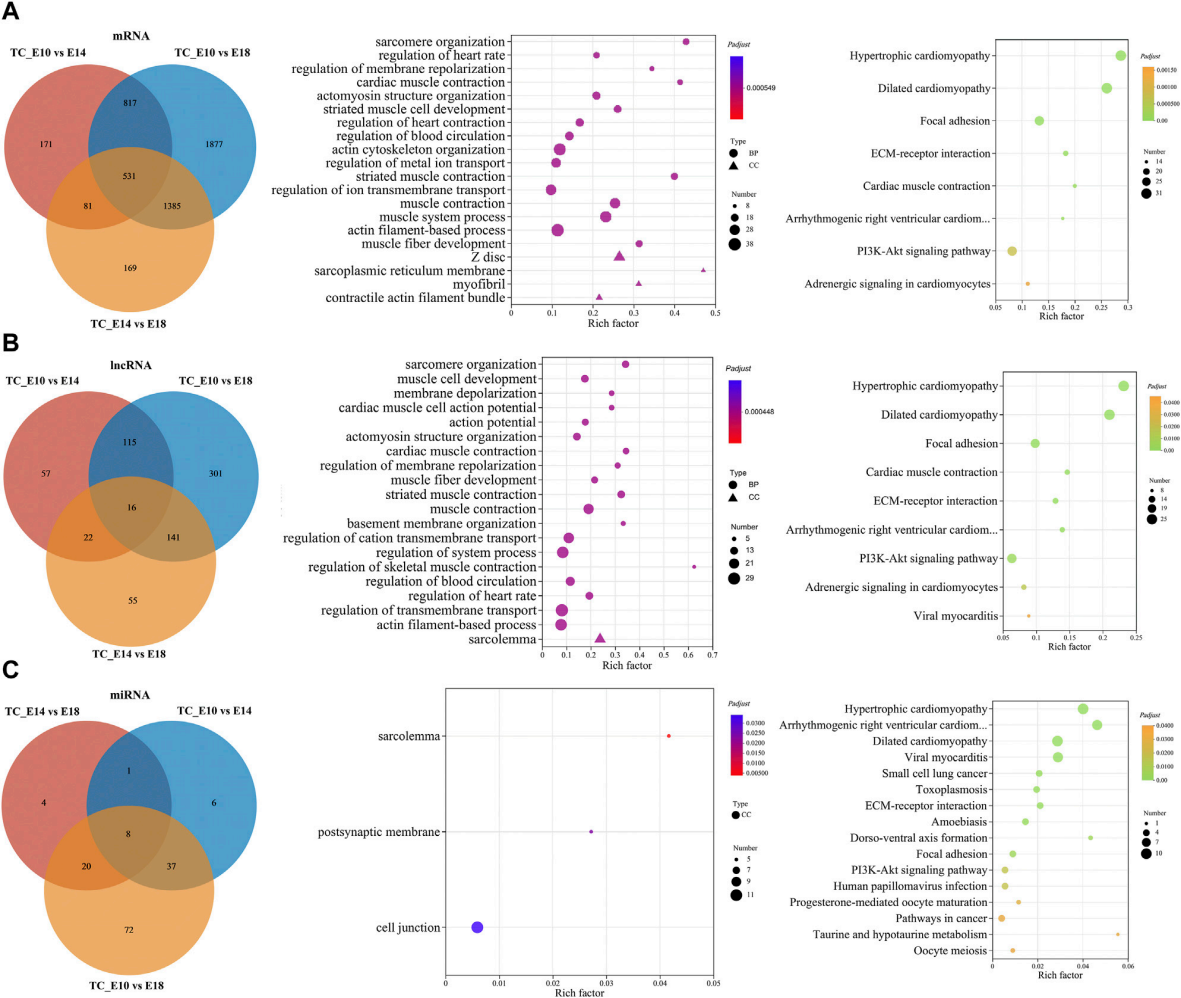
Functional prediction of common DE RNAs. Venn diagram of the number of common DE mRNAs, DE lncRNAs, and DE miRNAs in the three comparisons. GO and KEGG enrichment analyses of common DE mRNAs **(A)**, target mRNAs of common DE lncRNAs **(B)**, and DE miRNAs **(C)**. GO and KEGG bubble charts show the significantly enriched top 20 terms and pathways.

To further explore the regulation mechanisms of coding and non-coding RNAs in chicken leg muscle growth and development, the common DE RNAs that were differentially expressed in all three comparisons (E10 vs. E14, E10 vs. E18, and E14 vs. E18) were screened by Venn analysis. Therefore, 531 common DE mRNAs, 16 common DE lncRNAs, and eight common DE miRNAs were obtained (Figures 5A–C; Supplementary Data S11). Subsequently, we performed GO and KEGG enrichment analyses for these common DE mRNAs and target DE mRNAs during leg muscle growth and development in Tibetan chickens. For common DE mRNAs, target mRNAs of common DE lncRNAs, and common DE miRNAs, a total of 129, 118, and 3 GO terms and 8, 9, and 16 KEGG pathways were significantly enriched, respectively (Supplementary Data S12).

The GO enrichment analysis for common DE mRNAs suggested that significantly enriched top 20 GO terms mainly included sarcomere organization, actomyosin structure organization, striated muscle cell development, actin cytoskeleton organization, muscle system process, muscle fiber development, Z disc, myofibril, and muscle contraction. The KEGG enrichment analysis revealed only eight significant KEGG pathways, which mainly included focal adhesion, ECM–receptor interaction, PI3K-Akt signaling pathway, and other pathways related to cardiac muscle (Figure 5A).

The target mRNAs of common DE lncRNAs and DE miRNAs were predicted from the three comparisons. Significantly enriched top 20 GO terms for target mRNAs of common DE lncRNAs were primarily involved in sarcomere organization, muscle cell development, actomyosin structure organization, muscle fiber development, muscle contraction, regulation of skeletal muscle contraction, actin filament-based process, and sarcolemma. Furthermore, the significantly enriched KEGG pathways were similar to the pathways of common DE mRNAs (Figure 5B). The target mRNAs of common DE miRNAs significantly enriched only three GO terms: sarcolemma, postsynaptic membrane, and cell junction. Significantly enriched KEGG pathways included regulation of cardiac muscle, regulation of some diseases and cancer, ECM–receptor interaction, dorso-ventral axis formation, PI3K-Akt signaling pathway, progesterone-mediated oocyte maturation, taurine and hypotaurine metabolism, and oocyte meiosis (Figure 5C).

### 3.6 Construction of the ceRNA regulatory network related to muscle growth and development

DE mRNAs were selected from the significantly enriched GO terms associated with muscle growth and development. DE lncRNAs and DE miRNAs related to muscle growth and development were obtained through targeted relationships. Based on the three comparisons (E10 vs. E14, E10 vs. E18, and E14 vs. E18), we constructed three ceRNA networks related to muscle growth and development. In the comparison group E10 vs. E14, 205 DE mRNAs, 41 DE miRNAs, and 100 DE lncRNAs together constitute 370 lncRNA–miRNA–mRNA interaction pairs (Supplementary Figure S1; Supplementary Data S13). A total of 2,594 lncRNA–miRNA–mRNA interaction pairs were obtained in the ceRNA network of the comparison group E10 vs. E18 constructed with 936 DE mRNAs, 131 DE miRNAs, and 375 DE lncRNAs (Supplementary Figure S2; Supplementary Data S14). In the comparison group E14 vs. E18, the ceRNA network was constructed from 49 DE mRNAs, 19 DE miRNAs, and 53 DE mRNAs that yielded 78 lncRNA–miRNA–mRNA interaction pairs (Supplementary Figure S3; Supplementary Data S15). Considering that these ceRNA networks contain a large amount of information and each relationship cannot be shown in these figures, we constructed a mini-ceRNA network of common DE RNAs.

**FIGURE 6:**
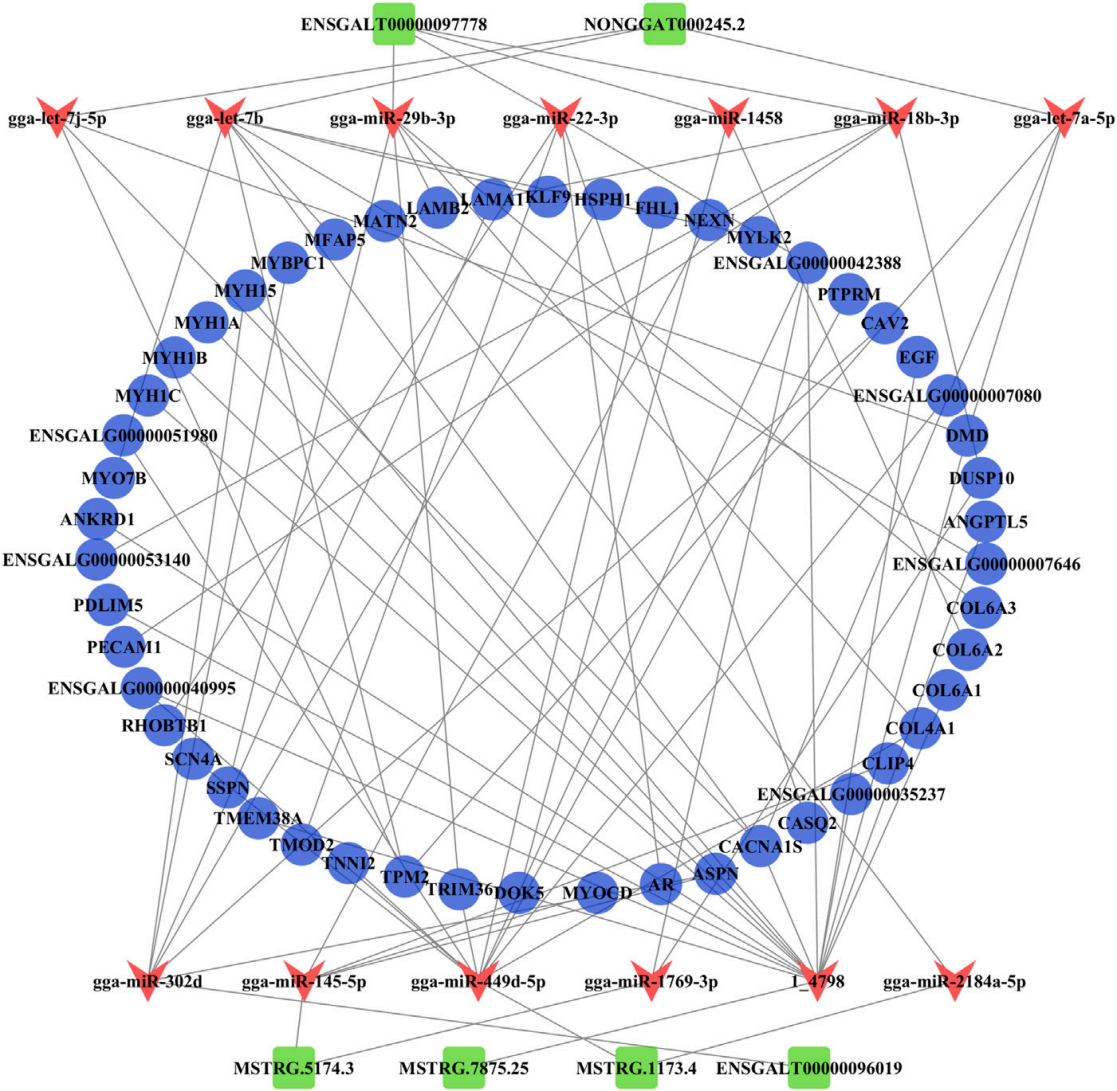
Analysis of the ceRNA (lncRNA–miRNA–mRNA) regulatory network. The blue circle, red V, and green rectangle notes represent the common DE mRNAs, DE miRNAs, and common DE lncRNAs, respectively.

The ceRNA regulatory network related to muscle growth and development was constructed from 50 common DE mRNAs, 13 DE miRNAs (gga-miR-1458, gga-let-7b, gga-miR-1769-3p, gga-miR-145-5p, gga-let-7j-5p, gga-miR-18b-3p, gga-let-7a-5p, gga-miR-29b-3p, gga-miR-22-3p, gga-miR-302d, gga-miR-2184a-5p, gga-miR-449d-5p, and 1_4798), and six common DE lncRNAs (ENSGALT00000097778, NONGGAT000245.2, MSTRG.5174.3, ENSGALT00000096019, MSTRG.1173.4, and MSTRG.7875.25) that yielded 67 pairs of candidate ceRNAs (lncRNA–miRNA–mRNA) (Figure 6; Supplementary Data S16).

### 3.7 Validation of RNA-seq results by RT-qPCR

**FIGURE 7:**
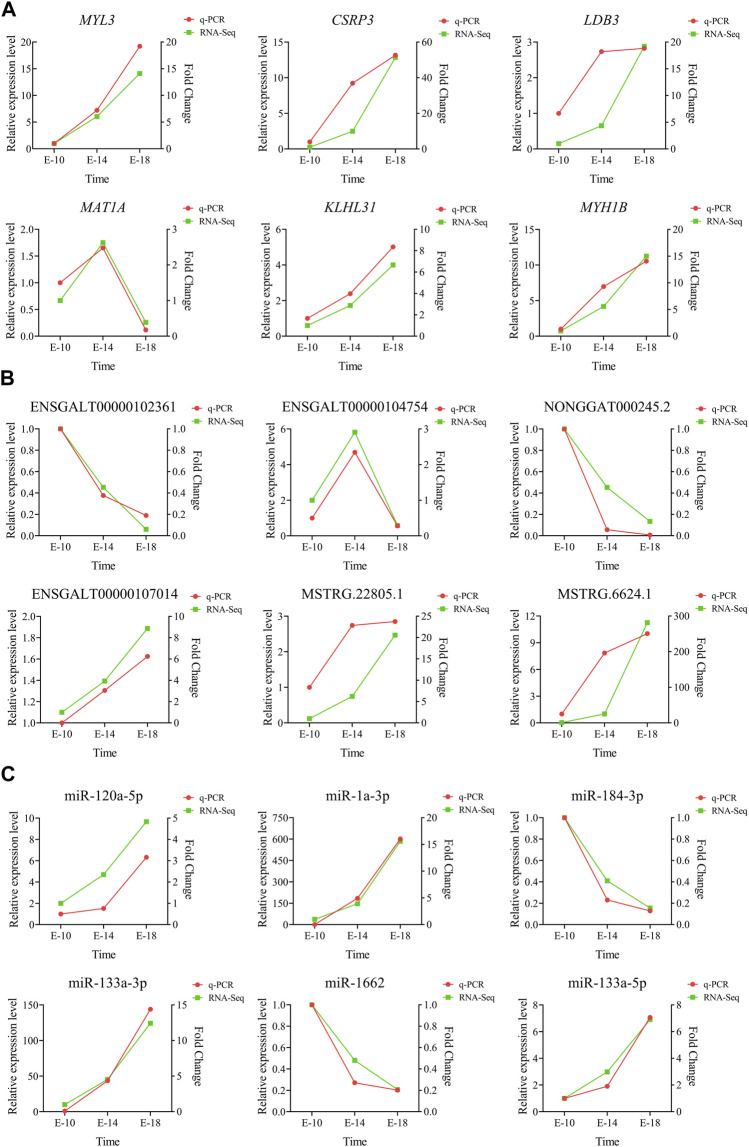
Validation of the RNA-seq data using RT-qPCR. The expression levels of six DE mRNAs **(A)**, six DE lncRNAs **(B)**, and six DE miRNAs **(C)** were validated with RT-qPCR in Tibetan chicken embryo leg muscles at three developmental stages.

The relative expression levels of six DE mRNAs (*MYL3*, *CSRP3*, *LDB3*, *MAT1A*, *KLHL31*, and *MYH1B*) (Figure 7A), six DE lncRNAs (ENSGALT00000102361, ENSGALT00000104754, NONGGAT000245.2, ENSGALT00000107014, MSTRG.22805.1, and MSTRG.6624.1) (Figure 7B), and six DE miRNAs (miR-120a-5p, miR-1a-3p, miR-184-3p, miR-133a-3p, miR-1662, and miR-133a-5p) (Figure 7C) were measured using RT-qPCR to validate the accuracy and reliability of the RNA-seq results. The results suggested that the expression trend of RT-qPCR for 18 DE RNAs at three developmental stages was generally consistent with the RNA-seq results. The aforementioned analysis indicated that the results of RNA-seq were reliable.

## 4 Discussion

Over the past 50 years, chickens have become the most consumed meat in the world (Petracci and Cavani, 2012). Skeletal muscle is one of the most important components of the biological body, accounting for approximately 40% of the body weight (Güller and Russell, 2010). The growth and development of skeletal muscle have a significant impact on the yield of livestock and poultry meat products (Berri et al., 2007). Therefore, it is of great significance to conduct research on skeletal muscle growth and development in livestock and poultry. In this study, we selected three stages (E10, E14, and E18), which were important for chicken skeletal muscle, to explore the molecular mechanisms of muscle growth and development in chickens. The expression profiles of mRNAs, lncRNAs, and miRNAs at the three stages were detected by RNA-seq and bioinformatics analysis. The potential functions of DE mRNAs and target mRNAs of DE lncRNAs and DE miRNAs in the three comparisons (E10 vs. E14, E10 vs. E18, and E14 vs. E18) were revealed. Then, we constructed the ceRNA regulatory network related to chicken skeletal muscle growth and development using the common DE mRNAs, DE lncRNAs, and DE miRNAs. In summary, our data provided a comprehensive understanding of the molecular regulatory mechanisms underlying skeletal muscle growth and development in chickens.

For the GO enrichment analysis of three comparisons, there were many GO terms related to skeletal muscle growth and development and limb formation in the comparison group E10 vs. E14, such as forelimb morphogenesis, sarcomere organization, muscle structure development, striated muscle cell development, and embryonic limb morphogenesis. In addition, many target mRNAs of DE lncRNAs were enriched in GO terms such as myofibril assembly, muscle fiber development, striated muscle contraction, M band, and muscle cell development. Many target mRNAs of DE miRNAs were enriched in the GO terms related to morphogenesis; the reason could be that during embryonic muscle development, the myogenic progenitor cells derived from the myotome continue to migrate and proliferate to the limbs, thereby promoting limb morphogenesis (Shi et al., 2022). These results suggested that the muscle had changed dramatically at E14 compared to E10 after continuous growth and development. Meanwhile, lncRNAs and miRNAs were extensively involved in muscle growth and development. Compared with the comparison group E10 vs. E14, the DE mRNAs and the target mRNAs of DE lncRNAs in the comparison groups E10 vs. E18 and E14 vs. E18 were mainly enriched in the GO terms associated with energy metabolism and mitochondrial electron transport chain besides the GO terms related to muscle growth and development. At the late stage of incubation, chicken embryos had frequent physiological activities and huge energy consumption. The mitochondrial electron transport chain is a metabolic pathway for organic molecules in organisms that helps them obtain energy (Kogot-Levin and Saada, 2014). Approximately 95% of the energy required for cellular activity comes from mitochondria (Chance et al., 1979). The growth and development of skeletal muscle were closely related to energy metabolism (Krischek et al., 2016). Some studies reported that insufficient energy could lead to weight loss in chickens and affect growth performance and muscle production in broilers (Nyo and Uni, 2010; Lamot et al., 2014).

For the KEGG enrichment analysis of three comparisons, ECM–receptor interaction, PI3K-Akt signaling pathway, TCA cycle, and glycolysis/gluconeogenesis were the most frequent pathways among all significantly enriched KEGG analyses. The main components of the ECM mainly included collagen, proteoglycan, and elastin (Yue, 2014). Apart from maintaining tissue structure, laminin in ECM was essential for early embryonic development and organogenesis (Rasmussen and Karsdal, 2019; Di Caprio and Bellas, 2020). The ECM had a beneficial effect on muscle cell differentiation, and ECM proteins (specifically, laminin and entactin) could enhance myotube formation (Grefte et al., 2016). It was shown that in the absence of an ECM–cellular receptor interaction signal, the expression of myogenin could not drive skeletal muscle differentiation (Osses and Brandan, 2002). In addition, the ECM also promoted myogenic differentiation and maturation (Yang et al., 2016). The PI3K-Akt pathway was involved in various life activities of the organism, such as cell cycle and cell apoptosis, lipid metabolism, glucose homeostasis, and protein synthesis (Wang et al., 2016; Zhu et al., 2021). Several studies found that the PI3K-Akt pathway played a crucial role in myoblast proliferation and differentiation by mediating transduction of many growth factors (Ling et al., 2021; Lyu et al., 2021; Oh et al., 2021). For example, miR-9-5p inhibited the proliferation and differentiation of chicken skeletal muscle satellite cells by targeting *IGF2BP3* (insulin-like growth factor-2 mRNA-binding protein 3) via the IGF2-PI3K/Akt signaling pathway (Yin et al., 2020). miR-485-5p had also been indicated to promote sheep myoblast proliferation by targeting *PPP1R13B* (protein phosphatase 1 regulatory subunit 13B) via the PI3K-Akt signaling pathway (Liu et al., 2023). Except for the mitochondrial electron transport chain, the pathways of TCA cycle and glycolysis/gluconeogenesis are also the energy sources for skeletal muscle growth, development, and activity. They were present in the E10 vs. E18 and E14 vs. E18 comparison groups, which suggested the energy requirements increased as the skeletal muscle continued to develop and mature. Interestingly, many pathways related to disease occurrence were enriched, which may help us better understand the connections between coding and non-coding RNAs and these diseases. GO and KEGG enrichment analyses demonstrated that the skeletal muscle of Tibetan chickens showed significantly different degrees of development at three different stages. These results showed that there were many coding and non-coding RNAs involved in skeletal muscle growth and development and provided a reference for understanding the overall view of the skeletal muscle growth and development process in Tibetan chickens.

To further explore the RNAs associated with skeletal muscle growth and development, we screened 531 common DE mRNAs, 16 common DE lncRNAs, and eight common DE miRNAs that were differentially expressed in all three comparisons. Subsequently, GO and KEGG enrichment analyses were performed for common DE mRNAs, target mRNAs of common DE lncRNAs, and DE miRNAs. As expected, plenty of GO terms related to muscle growth and development were significantly enriched. Similar to the aforementioned KEGG enrichment results, ECM–receptor interaction, PI3K-Akt signaling pathway, and several pathways linked with disease occurrence were mainly enriched. Therefore, numerous common DE mRNAs, common DE lncRNAs, and DE miRNAs regulated the growth and development of skeletal muscle. The ceRNA regulatory network is centered on miRNAs, linking mRNAs with lncRNAs/circRNAs to form a network of mutual influence and regulation (Poliseno et al., 2010; Salmena et al., 2011). Recently, numerous studies have shown that lncRNA–miRNA–mRNA is involved in the regulation of skeletal muscle growth and development (Cui et al., 2022; Shen et al., 2023). Therefore, we constructed a ceRNA regulatory network associated with skeletal muscle growth and development to better clarify the molecular mechanisms underlying skeletal muscle growth and development in chickens.

From the ceRNA (lncRNA–miRNA–mRNA) regulatory network, we found six common DE lncRNAs, including downregulated lnc-45.2 (NONGGAT000245.2), upregulated first and then downregulated lnc-778 (ENSGALT00000097778), and upregulated lnc-74.3 (MSTRG.5174.3), lnc-75.25 (MSTRG.7875.25), lnc-73.4 (MSTRG.1173.4), and lnc-019 (ENSGALT00000096019). lnc-45.2 could combine with the gga-let-7 family (gga-let-7b, gga-let-7j-5p, and gga-let-7a-5p). Lee et al. (2015) found that gga-let-7 family members were widely expressed during early chicken embryo differentiation, which indicated that gga-let-7 family members were important for controlling the differentiation process. Here, all gga-let-7 family members targeted *TPM2* (tropomyosin 2) and *CACNA1S* (calcium voltage-gated channel subunit alpha 1 S). The *TPM2* gene produced the Tpm2.2 protein isoform that played a regulatory role in the contractile thin filaments (Śliwinska et al., 2021). C*ACNA1S* was a key gene that regulated calcium ions. Previous studies have found that *CACNA1S* is related to muscle development, meat quality, and slaughter traits in livestock and poultry (Liu et al., 2018; Ren et al., 2021). Similarly, *CACNA1S* played a role in the regulation of skeletal muscle growth and development, such as muscle contraction, muscle system process, T-tubule, and I band, in this study. So, in the ceRNA network, the lnc-45.2-gga-let-7-*TPM2* and lnc-45.2-gga-let-7-*CACNA1S* interaction pairs may be involved in the formation of myofibril and sarcomere and regulate muscle contraction. lnc-778 showed a trend of upregulation and then downregulation in the three developmental stages of chickens, which indicated that skeletal muscle growth and development were extremely complex processes. It could bind to multiple miRNAs (gga-miR-1458, gga-miR-18b-3p, gga-miR-29b-3p, and gga-miR-22-3p). It had been reported that gga-miR-22-3p inhibited proliferation and promoted differentiation of skeletal muscle cells in porcine (Dang et al., 2020), sheep (Wang et al., 2022b), and C2C12 cells (Wang et al., 2018). The ceRNA network analysis showed that lnc-778 acted as a sponge for gga-miR-22-3p to increase the expression of *CASQ* (calsequestrin 1) and *AR* (androgen receptor) and suppress the expression of *RHOBTB1* (Rho-related BTB domain containing 1). Some studies suggested that *CASQ* was involved in muscle growth and development (Hou et al., 2021; Yang et al., 2021). *AR* affected the growth and development of sarcomere myofibrillar organization (Ghaibour et al., 2023). *RHOBTB1* has a function in actin filament system organization and cell proliferation regulation (Xiao et al., 2017; Silva et al., 2020). Therefore, the regulation of skeletal muscle cell proliferation and differentiation may be the result of the co-regulation of three interaction pairs composed of lnc-778, gga-miR-22-3p, *CASQ*, *AR*, and *RHOBTB1* in chickens. In our study, these miRNAs targeted many genes that were involved in the GO terms associated with sarcomere organization, muscle contraction, myofibril assembly, and regulation of the MAPK cascade.

For upregulated lncRNAs, lnc74.3 acted as a sponge for gga-miR-1769-3p to increase the expression of *ENSGALG00000042388* and *DUSP10* (dual specificity phosphatase 10), among which the *DUSP10* gene was a member of the dual-specificity MAPK phosphatase (DUSP) enzyme family. Various DUSP enzymes were vital in the regulation of mitogen-activated protein kinase (MAPK) signaling pathways; the activation states of MAPKs were primarily regulated by a family of DUSPs (Gém et al., 2021). Many studies found that MAPK signaling pathways contributed to muscle growth and development (Catterall, 2000; Dulhunty, 2006). Therefore, *DUSP10* may be involved in the regulation of skeletal muscle growth and development via the MAPK signaling pathway. lnc74.3 was also linked with *NEXN* (nexilin F-actin binding protein), *MYOCD* (myocardin), *CLIP4* (CAP-Gly domain containing linker protein family member 4), *ASPN* (asporin), and *CACNA1S* by targeting gga-miR-145-5p. *NEXN* was mainly related to actin binding, *MYOCD* was involved in the regulation of muscle contraction and muscle system processes, *CLIP4* was related to cytoskeletal development, and *ASPN* was involved in calcium ion binding and tissue development. lnc-019 could bind to gga-miR-302d, upregulating *CAV2* (caveolin 2), *MYBPC1* (myosin-binding protein C1), and *ASPN* and downregulating *HSPH1* (heat shock protein family H (Hsp110) member 1), *MYH15* (myosin heavy chain 15), and *LAMA1* (laminin subunit alpha 1). These mRNAs were all associated with muscle growth and development. For example, the myosin-binding protein C translated by *MYBPC1* was present in the A band of the sarcomere, regulating the cycling of actomyosin cross-bridges during muscle fiber contraction by interacting with the thick and thin filaments of the sarcomere (Geist and Kontrogianni, 2016). It is suggested that *MYBPC1* affects skeletal muscle satellite cell proliferation and differentiation (Velleman et al., 2021). Therefore, lnc-019 may regulate skeletal muscle growth and development through lnc-019–gga-miR-302d–*MYBPC1*. It is worth mentioning that other DE lncRNAs, including lnc-73.4, lnc-778, and lnc-75.25, which were involved in the ceRNA regulatory network, also played important roles in the regulation of skeletal muscle growth and development, except for the previously mentioned ceRNA regulation modalities. As a ceRNA for gga-miR-2184a-5p and gga-miR-449d-5p, lnc-73.4 regulated the expression of *MATN2* (matrilin 2) and *TNNI2* (troponin I2, fast skeletal type). *MATN2* played an important role in the formation of primary and secondary myofibers (Yuan et al., 2021). Nihashi et al. (2019) selected *TNNI2* as a candidate gene regulating myoblast proliferation and differentiation in chickens. Later, Yoshimoto et al. (2020) found that the expression of *TNNI2* gradually increased with muscle regeneration. Finally, lnc-75.25 acted as a “sponge” for the novel miRNA 1_4798, which could target 14 genes. According to the GO enrichment analysis, these genes were mainly related to the regulation of actomyosin, striated muscle, myofibril development, and regulation of calcium ion transport and binding, and most genes were upregulated. We speculated that this novel miRNA may be involved in skeletal muscle growth and development by regulating gene expression.

In summary, we constructed a ceRNA network that functions in chicken skeletal muscle. In the regulatory process, many lncRNAs acted as ceRNAs competing with mRNAs to bind miRNAs, indirectly regulating the expression levels of mRNAs related to skeletal muscle growth and development and ultimately affecting skeletal muscle growth and development, such as lnc-778–gga-miR-22-3p–CASQ, lnc-778–gga-miR-22-3p–AR, lnc-778–gga-miR-22-3p–RHOBTB1, lnc74.3–gga-miR-1769-3p–DUSP10, and lnc-019–gga-miR-302d–MYBPC. However, the biological function of the lncRNA–miRNA–mRNA interactions mentioned in this study necessitated further validation.

## 5 Conclusion

In this study, we used RNA-seq to systematically analyze the expression of mRNAs, lncRNAs, and miRNAs in the embryonic leg muscles of Tibetan chickens at three developmental stages. The functions of DE mRNAs, DE lncRNAs, and DE miRNAs in three comparisons were annotated by GO and KEGG enrichment analyses. The expression levels of various RNAs during skeletal muscle growth and development in chicken embryos were comprehensively revealed, which provided a genomic resource for a better understanding of the molecular mechanisms underlying skeletal muscle growth and development in the embryonic stage of chickens. In addition, we constructed a ceRNA network related to skeletal muscle growth and development and revealed several candidate lncRNA–miRNA–mRNA interaction pairs that may regulate skeletal muscle growth in chickens. However, the specific role of these candidate RNAs needs to be further validated. These results provide a theoretical basis for further studies on the mechanisms underlying skeletal muscle growth and development in chickens.

## Data Availability

The datasets presented in this study can be found in online repositories. The name of the repository/repositories and accession number(s) can be found below: https://www.ncbi.nlm.nih.gov/, No. PRJNA758717 and PRJNA954989.

## References

[B1] Berri C. Le Bihan-Duval E. Debut M. Santé-Lhoutellier V. Baéza E. Gigaud V. (2007). Consequence of muscle hypertrophy on characteristics of Pectoralis major muscle and breast meat quality of broiler chickens. J. Anim. Sci. 85, 2005–2011. 10.2527/jas.2006-398 17431054

[B2] Betel D. Wilson M. Gabow A. Marks D. S. Sander C. (2008). The microRNA.org resource: Targets and expression. Nucleic Acids Res. 36, D149–D153. 10.1093/nar/gkm995 18158296 PMC2238905

[B3] Braga E. A. Fridman M. V. Moscovtsev A. A. Filippova E. A. Dmitriev A. A. Kushlinskii N. E. (2020). LncRNAs in ovarian cancer progression, metastasis, and main pathways: ceRNA and alternative mechanisms. Int. J. Mol. Sci. 21, 8855. 10.3390/ijms21228855 33238475 PMC7700431

[B4] Cai B. L. Li Z. H. Ma M. T. Wang Z. J. Han P. G. Abdalla B. A. (2017). LncRNA-Six1 encodes a micropeptide to activate Six1 in cis and is involved in cell proliferation and muscle growth. Front. Physiol. 8, 230. 10.3389/fphys.2017.00230 28473774 PMC5397475

[B5] Catterall W. A. (2000). Structure and regulation of voltage-gated Ca^2+^ channels. Annu. Rev. Cell. Dev. Biol. 16, 521–555. 10.1146/annurev.cellbio.16.1.521 11031246

[B6] Chance B. Sies H. Boveris A. (1979). Hydroperoxide metabolism in mammalian organs. Physiol. Rev. 59, 527–605. 10.1152/physrev.1979.59.3.527 37532

[B7] Chen W. Lv Y. T. Zhang H. X. Ruan D. Wang S. Lin Y. C. (2013). Developmental specificity in skeletal muscle of late-term avian embryos and its potential manipulation. Poult. Sci. 92, 2754–2764. 10.3382/ps.2013-03099 24046424

[B8] Chi Y. D. Xu Y. U. Luo F. Lin Y. Q. Li Z. X. (2020). Molecular cloning, expression profiles and associations of KLF6 gene with intramuscular fat in Tibetan chicken. Anim. Biotechnol. 31, 67–75. 10.1080/10495398.2018.1540428 30501383

[B9] Cui R. Kang X. L. Liu Y. F. Liu X. M. Chan S. H. Wang Y. B. (2022). Integrated analysis of the whole transcriptome of skeletal muscle reveals the ceRNA regulatory network related to the formation of muscle fibers in Tan sheep. Front. Genet. 13, 991606. 10.3389/fgene.2022.991606 36330447 PMC9624228

[B10] Dang H. Q. Xu G. L. Hou L. J. Xu J. Hong G. L. Hu C. Y. (2020). MicroRNA-22 inhibits proliferation and promotes differentiation of satellite cells in porcine skeletal muscle. J. Integ. Agr. 19 (1), 225–233. 10.1016/s2095-3119(19)62701-2

[B11] Di Caprio N. Bellas E. (2020). Collagen stiffness and architecture regulate fibrotic gene expression in engineered adipose tissue. Adv. Biosyst. 4, e1900286. 10.1002/adbi.201900286 32529801

[B12] Dong X. X. Cheng Y. Qiao L. Y. Wang X. Zeng C. P. Feng Y. P. (2021). Male-biased gga-miR-2954 regulates myoblast proliferation and differentiation of chicken embryos by targeting YY1. Genes (Basel) 12, 1325. 10.3390/genes12091325 34573307 PMC8470131

[B13] Dransfield E. Sosnicki A. A. (1999). Relationship between muscle growth and poultry meat quality. Poult. Sci. 78, 743–746. 10.1093/ps/78.5.743 10228972

[B14] Dulhunty A. F. (2006). Excitation-contraction coupling from the 1950s into the new millennium. Clin. Exp. Pharmacol. Physiol. 3, 763–772. 10.1111/j.1440-1681.2006.04441.x 16922804

[B15] Friedländer M. R. Mackowiak S. D. Li N. Chen W. Rajewsky N. (2012). miRDeep2 accurately identifies known and hundreds of novel microRNA genes in seven animal clades. Nucleic Acids Res. 40, 37–52. 10.1093/nar/gkr688 21911355 PMC3245920

[B16] Geist J. Kontrogianni K. A. (2016). MYBPC1, an emerging myopathic gene: What we know and what we need to learn. Front. Physiol. 7, 410. 10.3389/fphys.2016.00410 27683561 PMC5021714

[B17] Gém J. B. Kovács K. B. Szalai L. Szakadáti G. Porkoláb E. Szalai B. (2021). Characterization of type 1 angiotensin II receptor activation induced dual-specificity MAPK phosphatase gene expression changes in rat vascular smooth muscle cells. Cells 10, 3538. 10.3390/cells10123538 34944046 PMC8700539

[B18] Ghaibour K. Schuh M. Souali-Crespo S. Chambon C. Charlot A. Rizk J. (2023). Androgen receptor coordinates muscle metabolic and contractile functions. J. Cachexia Sarcopenia Muscle 2023, 13251. 10.1002/jcsm.13251 PMC10401547 37208984

[B19] Grefte S. Adjobo-Hermans M. J. W. Versteeg E. M. M. Koopman W. J. H. Daamen W. F. (2016). Impaired primary mouse myotube formation on crosslinked type I collagen films is enhanced by laminin and entactin. Acta. Biomater. 30, 265–276. 10.1016/j.actbio.2015.11.009 26555376

[B20] Güller I. Russell A. P. (2010). MicroRNAs in skeletal muscle: Their role and regulation in development, disease and function. J. Physiol. 588, 4075–4087. 10.1113/jphysiol.2010.194175 20724363 PMC3002442

[B21] Guttman M. Rinn J. L. (2012). Modular regulatory principles of large non-coding RNAs. Nature 482, 339–346. 10.1038/nature10887 22337053 PMC4197003

[B22] Hou H. Wang X. Yang C. Cai X. Lv W. Tu Y. (2021). Comparative genome and transcriptome integration studies reveal the mechanism of pectoral muscle development and function in pigeons. Front. Genet. 12, 735795. 10.3389/fgene.2021.735795 34987544 PMC8721168

[B23] Kim D. Paggi J. M. Park C. Bennett C. Salzberg S. L. (2019). Graph-based genome alignment and genotyping with HISAT2 and HISAT-genotype. Nat. Biotechnol. 37, 907–915. 10.1038/s41587-019-0201-4 31375807 PMC7605509

[B24] Kogot-Levin A. Saada A. (2014). Ceramide and the mitochondrial respiratory chain. Biochimie 100, 88–94. 10.1016/j.biochi.2013.07.027 23933096

[B25] Kong L. Zhang Y. Ye Z. Q. Liu X. Q. Zhao S. Q. Wei L. P. (2007). CPC: Assess the protein-coding potential of transcripts using sequence features and support vector machine. Nucleic Acids Res. 35, 345–349. 10.1093/nar/gkm391 PMC1933232 17631615

[B26] Krischek C. Janisch S. Naraballobh W. Brunner R. Wimmers K. Wicke M. (2016). Altered incubation temperatures between embryonic Days 7 and 13 influence the weights and the mitochondrial respiratory and enzyme activities in breast and leg muscles of broiler embryos. Mol. Reprod. Dev. 83, 71–78. 10.1002/mrd.22596 26599350

[B27] Lamot D. M. van de Linde I. B. Molenaar R. van der Pol C. W. Wijtten P. J. Kemp B. (2014). Effects of moment of hatch and feed access on chicken development. Poult. Sci. 93, 2604–2614. 10.3382/ps.2014-04123 25071231

[B28] Langmead B. Salzberg S. L. (2012). Fast gapped-read alignment with Bowtie 2. Nat. Methods. 9, 357–359. 10.1038/nmeth.1923 22388286 PMC3322381

[B29] Lee S. I. Jeon M. H. Kim J. S. Jeon I. S. Byun S. J. (2015). The gga-let-7 family post-transcriptionally regulates TGFBR1 and LIN28B during the differentiation process in early chick development. Mol. Reprod. Dev. 82, 967–975. 10.1002/mrd.22575 26297836

[B30] Lewis B. P. Shih I. H. Jones-Rhoades M. W. Bartel D. P. Burge C. B. (2003). Prediction of mammalian microRNA targets. Cell 115, 787–798. 10.1016/s0092-8674(03)01018-3 14697198

[B31] Li J. X. Zhao W. J. Li Q. Q. Huang Z. Y. Shi G. L. Li C. C. (2020). Long non-coding RNA H19 promotes porcine satellite cell differentiation by interacting with TDP43. Genes (Basel) 11, 259. 10.3390/genes11030259 32121115 PMC7140797

[B32] Li Y. Y. Chen X. N. Sun H. Wang H. T. (2018). Long non-coding RNAs in the regulation of skeletal myogenesis and muscle diseases. Cancer. Lett. 417, 58–64. 10.1016/j.canlet.2017.12.015 29253523

[B33] Ling M. F. Quan L. L. Lai X. M. Lang L. M. Li F. Yang X. H. (2021). VEGFB promotes myoblasts proliferation and differentiation through VEGFR1-PI3K/Akt signaling pathway. Int. J. Mol. Sci. 22, 13352. 10.3390/ijms222413352 34948148 PMC8707860

[B34] Liu M. Li B. Peng W. W. Ma Y. L. Huang Y. Z. Lan X. Y. (2019). LncRNA-MEG3 promotes bovine myoblast differentiation by sponging miR-135. J. Cell. Physil. 234, 18361–18370. 10.1002/jcp.28469 30887511

[B35] Liu S. Q. Liu Z. Y. Wang P. Li W. T. Zhao S. G. Liu Y. F. (2023). Estrogen-mediated oar-miR-485-5p targets PPP1R13B to regulate myoblast proliferation in sheep. Int. J. Biol. Macromol. 236, 123987. 10.1016/j.ijbiomac.2023.123987 36906210

[B36] Liu Y. H. Jia Y. X. Liu C. Ding L. M. Xia Z. F. (2018). RNA-Seq transcriptome analysis of breast muscle in Pekin ducks supplemented with the dietary probiotic Clostridium butyricum. BMC Genom 19 (1), 844. 10.1186/s12864-018-5261-1 PMC6264624 30486769

[B37] Love M. I. Huber W. Anders S. (2014). Moderated estimation of fold change and dispersion for RNA-seq data with DESeq2. Genome. Biol. 15, 550. 10.1186/s13059-014-0550-8 25516281 PMC4302049

[B38] Lyu M. Wang X. Meng X. Y. Qian H. R. Li Q. Ma B. X. (2021). chi-miR-487b-3p inhibits goat myoblast proliferation and differentiation by targeting IRS1 through the IRS1/PI3K/Akt signaling pathway. Int. J. Mol. Sci. 23, 115. 10.3390/ijms23010115 35008541 PMC8745444

[B39] Matsumoto A. Pasut A. Matsumoto M. Yamashita R. Fung J. Monteleone E. (2017). mTORC1 and muscle regeneration are regulated by the LINC00961-encoded SPAR polypeptide. Nature 541, 228–232. 10.1038/nature21034 28024296

[B40] Nihashi Y. Umezawa K. Shinji S. Hamaguchi Y. Kobayashi H. Kono T. (2019). Distinct cell proliferation, myogenic differentiation, and gene expression in skeletal muscle myoblasts of layer and broiler chickens. Sci. Rep. 9, 16527. 10.1038/s41598-019-52946-4 31712718 PMC6848216

[B41] Nyo Y. Uni Z. (2010). Early nutritional strategies World. Poult. Sci. J. 66, 639–646. 10.1017/s0043933910000620

[B42] Oh M. Kim S. Y. Park S. Kim K. N. Kim S. H. (2021). Phytochemicals in Chinese chive (Allium tuberosum) induce the skeletal muscle cell proliferation via PI3K/Akt/mTOR and smad pathways in C2C12 cells. Int. J. Mol. Sci. 22, 2296. 10.3390/ijms22052296 33669060 PMC7956299

[B43] Osses N. Brandan E. (2002). ECM is required for skeletal muscle differentiation independently of muscle regulatory factor expression. Am. J. Physiol. Cell. Physiol. 282, C383–C394. 10.1152/ajpcell.00322.2001 11788350

[B44] Pertea M. Pertea G. M. Antonescu C. M. Chang T. C. Mendell J. T. Salzberg S. L. (2015). StringTie enables improved reconstruction of a transcriptome from RNA-seq reads. Nat. Biotechnol. 33, 290–295. 10.1038/nbt.3122 25690850 PMC4643835

[B45] Petracci M. Cavani C. (2012). Muscle growth and poultry meat quality issues. Nutrients 4, 1–12. 10.3390/nu4010001 22347614 PMC3277097

[B46] Picard B. Berri C. Lefaucheur L. Molette C. Sayd T. Terlouw C. (2010). Skeletal muscle proteomics in livestock production. Brief. Funct. Genomics 9, 259–278. 10.1093/bfgp/elq005 20308039

[B47] Poliseno L. Salmena L. Zhang J. W. Carver B. Haveman W. J. Pandolfi P. P. (2010). A coding-independent function of gene and pseudogene mRNAs regulates tumour biology. Nature 465, 1033–1038. 10.1038/nature09144 20577206 PMC3206313

[B48] Rasmussen D. G. K. Karsdal M. A. (2019). Laminins Biochemistry of collagens, laminins and elastin. Second Edition. Pittsburgh, USA: Academic Press, 209–263. 10.1016/b978-0-12-817068-7.00029-x

[B49] Ren L. T. Liu A. F. Wang Q. G. Wang H. G. Dong D. Q. Liu L. B. (2021). Transcriptome analysis of embryonic muscle development in Chengkou Mountain Chicken. BMC Genom 22, 431. 10.1186/s12864-021-07740-w PMC8191012 34107874

[B50] Robert D. F. Alex B. Jody C. Penelope C. Ruth Y. E. Sean R. E. (2014). Pfam: The protein families database. Nucleic Acids Res. 42, 222–230. 10.1002/047001153x.g306303

[B51] Salmena L. Poliseno L. Tay Y. Kats L. Pandolfi P. P. (2011). A ceRNA hypothesis: The rosetta stone of a hidden RNA language? Cell 146, 353–358. 10.1016/j.cell.2011.07.014 21802130 PMC3235919

[B52] Shen J. Y. Luo Y. Z. Wang J. Q. Hu J. Liu X. Li S. B. (2023). Integrated transcriptome analysis reveals roles of long non-coding RNAs (lncRNAs) in caprine skeletal muscle mass and meat quality. Funct. Integr. Genomics 23, 63. 10.1007/s10142-023-00987-4 36810929

[B53] Shi H. M. He Y. Li X. Z. Du Y. L. Zhao J. B. Ge C. R. (2022). Regulation of non-coding RNA in the growth and development of skeletal muscle in domestic chickens. Genes (Basel) 13, 1033. 10.3390/genes13061033 35741795 PMC9222894

[B54] Silva D. B. S. Fonseca L. F. S. Pinheiro D. G. Magalhães A. F. B. Muniz M. M. M. Ferro J. A. (2020). Spliced genes in muscle from Nelore Cattle and their association with carcass and meat quality. Sci. Rep. 10, 14701. 10.1038/s41598-020-71783-4 32895448 PMC7477197

[B55] Śliwinska M. Robaszkiewicz K. Wasąg P. Moraczewska J. (2021). Mutations Q93H and E97K in TPM2 disrupt ca-dependent regulation of actin filaments. Int. J. Mol. Sci. 22, 4036. 10.3390/ijms22084036 33919826 PMC8070786

[B56] Stickland N. C. Bayol S. Ashton C. Rehfeldt C. (2004). “Manipulation of muscle fibre number during prenatal development,” in Muscle development of livestock animals: Physiology, genetics and meat quality (London, UK: CABI Books, CABI International), 69–82. 10.1079/9780851998114.0069

[B57] Stockdale F. E. Miller J. B. (1987). The cellular basis of myosin heavy chain isoform expression during development of avian skeletal muscles. Dev. Biol. 123, 1–9. 10.1016/0012-1606(87)90420-9 3305110

[B58] Sun L. Luo H. T. Bu D. C. Zhao G. G. Yu K. T. Zhang C. H. (2013). Utilizing sequence intrinsic composition to classify protein-coding and long non-coding transcripts. Nucleic Acids Res. 41, e166. 10.1093/nar/gkt646 23892401 PMC3783192

[B59] Tang J. Ge S. J. C. Tu X. L. Wang M. L. (2015). Specificity of Tibetan Chicken germplasm resources and its breeding and utilization in Shannan prefecture of Tibet. Animal Husb. Feed Sci. 3, 137–139+143. 10.13989/j.cnki.0517-6611.2014.29.042

[B60] Velleman S. G. Coy C. S. Abasht B. (2021). Effect of growth selection of broilers on breast muscle satellite cell function: Response of satellite cells to NOV, COMP, MYBP-C1, and CSRP3. Comp. Biochem. Physiol. A. Mol. Integr. Physiol. 255, 110917. 10.1016/j.cbpa.2021.110917 33548540

[B61] Wang H. Zhang Q. Wang B. B. Wu W. J. Wei J. L. Li P. H. (2018). miR-22 regulates C2C12 myoblast proliferation and differentiation by targeting TGFBR1. J. Cell. Biol. 97, 257–268. 10.1016/j.ejcb.2018.03.006 29588073

[B62] Wang J. Chen M. Y. Chen J. F. Ren Q. L. Zhang J. Q. Cao H. (2020). LncRNA IMFlnc1 promotes porcine intramuscular adipocyte adipogenesis by sponging miR-199a-5p to up-regulate CAV-1. Bmc. Mol. Cell. Biol. 21, 77. 10.1186/s12860-020-00324-8 33148167 PMC7640402

[B63] Wang L. G. Park H. J. Dasari S. Wang S. Q. Kocher J. P. Li W. (2013). Cpat: Coding-potential assessment tool using an alignment-free logistic regression model. Nucleic Acids Res. 41, e74. 10.1093/nar/gkt006 23335781 PMC3616698

[B64] Wang R. Zhang Q. Peng X. Zhou C. Zhong Y. X. Chen X. (2016). Stellettin B induces G1 arrest, apoptosis and autophagy in human non-small cell lung cancer A549 cells via blocking PI3K/Akt/mTOR pathway. Sci. Rep. 6, 27071. 10.1038/srep27071 27243769 PMC4886687

[B65] Wang S. S. Cao X. K. Ge L. Gu Y. F. Lv X. Y. Getachew T. (2022b). MiR-22-3p inhibits proliferation and promotes differentiation of skeletal muscle cells by targeting IGFBP3 in Hu sheep. Anim. (Basel) 12, 114. 10.3390/ani12010114 PMC8749897 35011220

[B66] Wang S. S. Tan B. H. Xiao L. Y. Zeng J. K. Zhao X. M. Hong L. J. (2022a). Long non-coding RNA Gm10561 promotes myogenesis by sponging miR-432. Epigenetics 17, 2039–2055. 10.1080/15592294.2022.2105052 35899799 PMC9665130

[B67] Xiao J. J. Liu H. Cretoiu D. Toader D. O. Suciu N. Shi J. (2017). miR-31a-5p promotes postnatal cardiomyocyte proliferation by targeting RhoBTB1. Exp. Mol. Med. 49, e386. 10.1038/emm.2017.150 29053138 PMC5668467

[B68] Yang B. G. Yuan Y. Zhou D. K. Ma Y. H. Mahrous K. F. Wang S. Z. (2021). Genome-wide selection signal analysis of Australian Boer goat reveals artificial selection imprinting on candidate genes related to muscle development. Anim. Genet. 52, 550–555. 10.1111/age.13092 34029388

[B69] Yang H. S. Lee B. Tsui J. H. Macadangdang J. Jang S. Y. Im S. G. (2016). Electroconductive nanopatterned substrates for enhanced myogenic differentiation and maturation. Adv. Healthc. Mat. 5, 137–145. 10.1002/adhm.201500003 PMC5003176 25988569

[B70] Yin H. D. He H. R. Shen X. X. Zhao J. Cao X. A. Han S. S. (2020). miR-9-5p inhibits skeletal muscle satellite cell proliferation and differentiation by targeting IGF2BP3 through the IGF2-PI3K/Akt signaling pathway. Int. J. Mol. Sci. 21, 1655. 10.3390/ijms21051655 32121275 PMC7084337

[B71] Yoshimoto Y. Ikemoto U. M. Hitachi K. Fukada S. I. Uezumi A. (2020). Methods for accurate assessment of myofiber maturity during skeletal muscle regeneration. Front. Cell. Dev. Biol. 8, 267. 10.3389/fcell.2020.00267 32391357 PMC7188918

[B72] Yu X. Wang Z. Sun H. Yang Y. Li K. Tang Z. (2018). Long non-coding MEG3 is a marker for skeletal muscle development and meat production traits in pigs. Anim. Genet. 49, 571–578. 10.1111/age.12712 30294799

[B73] Yuan R. Q. Zhang J. M. Wang Y. J. Zhu X. X. Hu S. L. Zeng J. H. (2021). Reorganization of chromatin architecture during prenatal development of porcine skeletal muscle. DNA Res. 28, dsab003. 10.1093/dnares/dsab003 34009337 PMC8154859

[B74] Yue B. (2014). Biology of the extracellular matrix: An overview. J. Glaucoma 23, S20–S23. 10.1097/ijg.0000000000000108 25275899 PMC4185430

[B75] Zhan S. Y. Qin C. Y. Li D. D. Zhao W. Nie L. Cao J. X. (2019). A novel long noncoding RNA, lncR-125b, promotes the differentiation of goat skeletal muscle satellite cells by sponging miR-125b. Front. Genet. 10, 1171. 10.3389/fgene.2019.01171 31803241 PMC6872680

[B76] Zhan S. Y. Zhang Y. Yang C. T. Li D. D. Zhong T. Wang L. J. (2022). LncR-133a suppresses myoblast differentiation by sponging miR-133a-3p to activate the FGFR1/ERK1/2 signaling pathway in goats. Genes (Basel) 13, 818. 10.3390/genes13050818 35627202 PMC9141198

[B77] Zhang W. Z. Sun B. Zhao Y. Q. Raza S. H. A. Li Y. S. Wang J. F. (2022). Proliferation of bovine myoblast by LncPRRX1 via regulation of the miR-137/CDC42 axis. Int. J. Biol. Macromol. 220, 33–42. 10.1016/j.ijbiomac.2022.08.018 35944756

[B78] Zhang X. J. Chen M. M. Liu X. F. Zhang L. L. Ding X. B. Guo Y. W. (2020). A novel lncRNA, lnc403, involved in bovine skeletal muscle myogenesis by mediating KRAS/Myf6. Gene 751, 144706. 10.1016/j.gene.2020.144706 32387386

[B79] Zhu X. Ren L. Liu J. J. Chen L. Cheng J. Chu W. Y. (2021). Transcriptome analysis provides novel insights into the function of PI3K/AKT pathway in maintaining metabolic homeostasis of Chinese perch muscle. Aquacult. Rep. 21, 100838. 10.1016/j.aqrep.2021.100838

