# Supplementary material for: Transcriptome analysis of mRNAs, lncRNAs, and miRNAs in the skeletal muscle of Tibetan chickens at different developmental stages
Source: Front Physiol. 2023 Jul 26;14:1225349. doi: 10.3389/fphys.2023.1225349 (PMC10410567; doi:10.3389/fphys.2023.1225349)
Supplement: Supplementary file 1 [file DataSheet1.ZIP › Supplementary Figure S1-3.pdf]

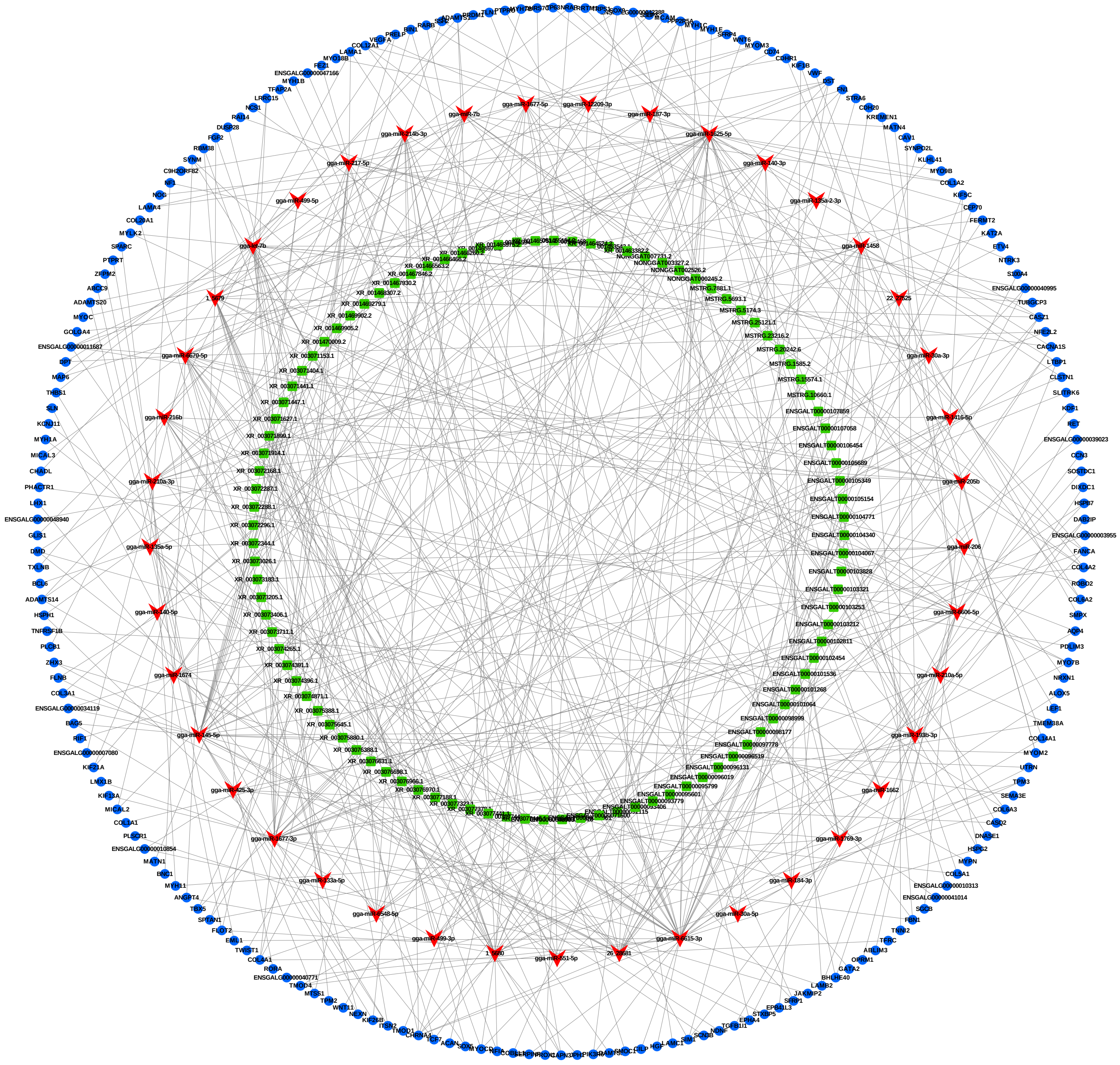

Supplemental Figure 1. The DE lncRNA-miRNA-mRNA interaction network related to muscle growth and development in E10 vs E14. The blue circle, red V, and green rectangle notes represent the DE mRNAs, DE miRNAs, and DE lncRNAs, respectively.

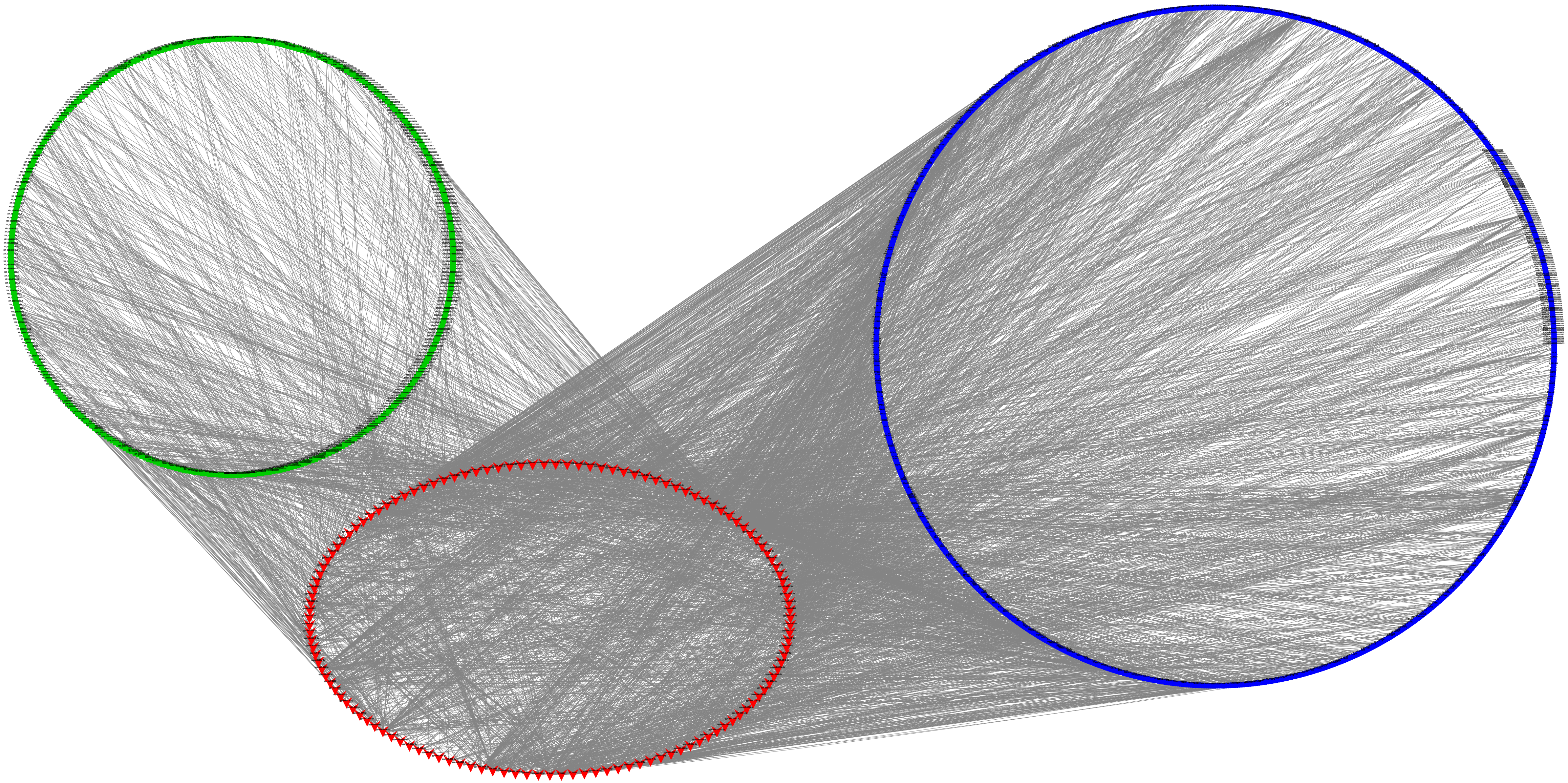

Supplemental Figure 2. The DE lncRNA-miRNA-mRNA interaction network related to muscle growth and development in E10 vs E18. The blue circle, red V, and green rectangle notes represent the DE mRNAs, DE miRNAs, and DE lncRNAs, respectively.

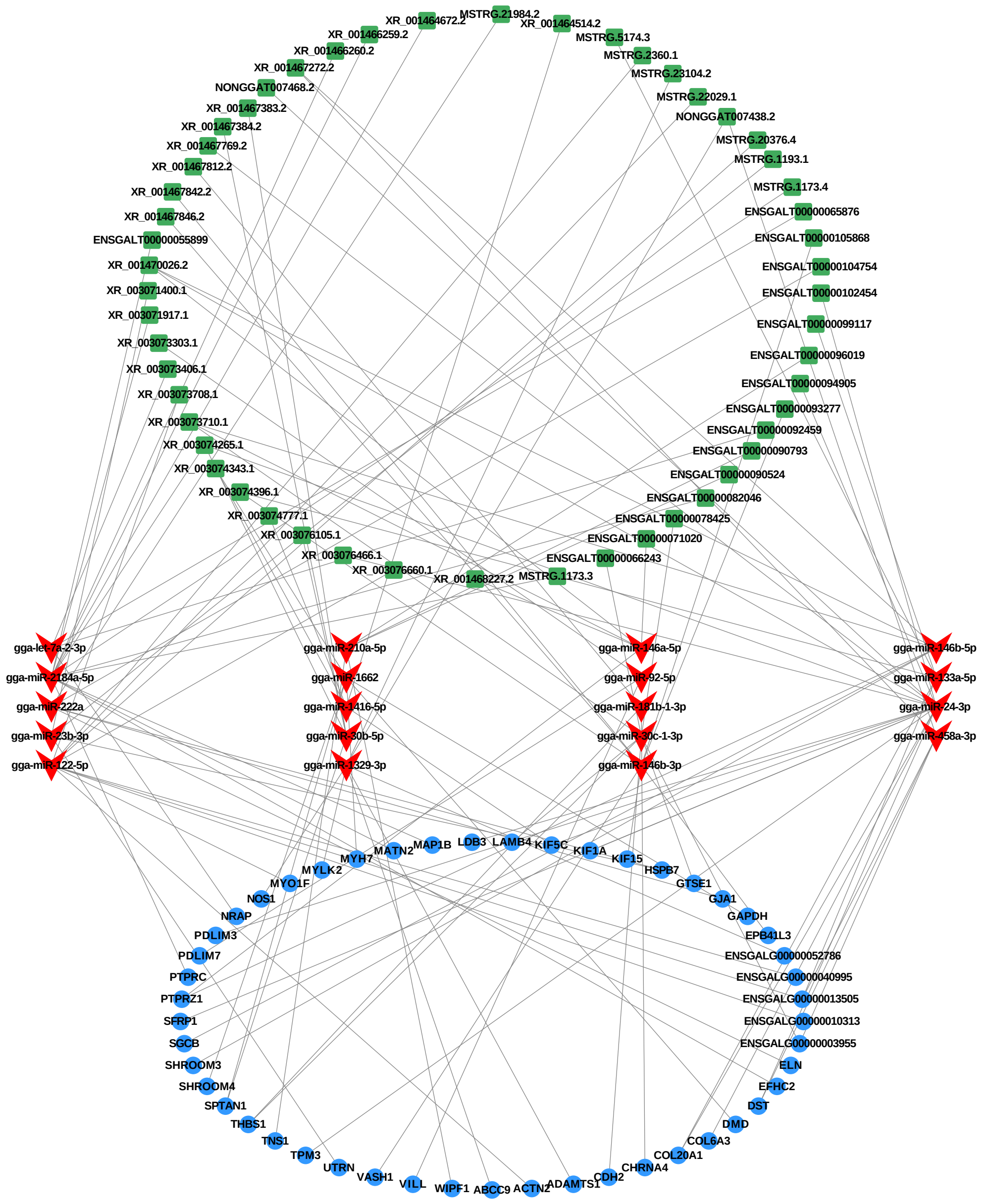

Supplemental Figure 3. The DE lncRNA-miRNA-mRNA interaction network related to muscle growth and development in E14 vsE18. The blue circle, red V, and green rectangle notes represent the DE mRNAs, DE miRNAs, and DE lncRNAs, respectively.
