# Supplementary material for: Transcriptome analysis of mRNAs, lncRNAs, and miRNAs in the skeletal muscle of Tibetan chickens at different developmental stages
Source: Front Physiol. 2023 Jul 26;14:1225349. doi: 10.3389/fphys.2023.1225349 (PMC10410567; doi:10.3389/fphys.2023.1225349)
Supplement: Supplementary file 2 [file Table1.DOC]

Supplementary Material

Transcriptome analysis of mRNAs, lncRNAs, and miRNAs in Skeletal Muscle of Tibetan Chickens at Different Developmental Stages

Jie Li^1,2^, Chuwen Chen^1,2^, Ruipeng Zhao^1,3^, Jinbo Wu^4^, and Zhixiong, Li^1,2*^

*** Correspondence:** Zhixiong, Li: lizhixiong@swun.edu.cn

# Supplementary Data

Supplementary Data S1: Primer information of RT-qPCR used in the study,

Supplementary Data S2: List of software and databases used in the study,

Supplementary Data S3: Data summary of RNA-seq in Tibetan chicken leg muscle,

Supplementary Data S4: The results of long RNA-seq mapping and small RNA-seq mapping,

Supplementary Data S5: List of DE mRNAs, DE lncRNAs, and DE miRNAs between E10 and E14,

Supplementary Data S6: List of the top 20 up-regulation and down-regulated lncRNA and miRNAs in E10 vs E14 based on log_2_(Fold Change),

Supplementary Data S7: List of DE mRNAs, DE lncRNAs, and DE miRNAs between E10 and E18,

Supplementary Data S8: List of the top 20 up-regulation and down-regulated lncRNA and miRNAs in E10 vs E18 based on log_2_(Fold Change),

Supplementary Data S9: List of DE mRNAs, DE lncRNAs, and DE miRNAs between E14 and E18,

Supplementary Data S10: List of the top 20 up-regulation and down-regulated lncRNA and miRNAs in E14 vs E18 based on log_2_(Fold Change),

Supplementary Data S11: List of common DE mRNAs, DE lncRNAs, and DE miRNAs,

Supplementary Data S12: The significantly enriched GO terms and KEGG pathways of common DE mRNAs and target mRNAs of common DE lncRNAs and common DE miRNAs,

Supplementary Data S13: List of DE lncRNA-miRNA-mRNA interaction pairs related to muscle growth and development in E10 vs E14,

Supplementary Data S14: List of DE lncRNA-miRNA-mRNA interaction pairs related to muscle growth and development in E10 vs E18,

Supplementary Data S15: List of DE lncRNA-miRNA-mRNA interaction pairs related to muscle growth and development in E14 vs E18,

Supplementary Data S16: List of common DE lncRNA-miRNA-mRNA interaction pairs related to muscle growth and development.

# Supplementary Figure

Supplementary Figure S1: The DE lncRNA-miRNA-mRNA interaction network related to muscle growth and development in E10 vs E14.

Supplementary Figure S2: The DE lncRNA-miRNA-mRNA interaction network related to muscle growth and development in E10 vs E18.

Supplementary Figure S3: The DE lncRNA-miRNA-mRNA interaction network related to muscle growth and development in E14 vsE18.
